# Social influence makes outlier opinions in online reviews offer more helpful information

**DOI:** 10.1038/s41598-023-35953-4

**Published:** 2023-06-27

**Authors:** Kunhao Yang, Itsuki Fujisaki, Kazuhiro Ueda

**Affiliations:** 1 Faculty of Law, Chuo Gakuin University, Abiko, Chiba 270-1196 Japan; 2 Graduate School of Arts and Sciences, The University of Tokyo, Tokyo, 153-8902 Japan; 3 Present Address: Graduate School of Science and Technology for Innovation, Yamaguchi University, Ube, Yamaguchi 755-0018 Japan; 4 Present Address: Graduate School of Information Sciences, Tohoku University, Sendai, Miyagi 980-0845 Japan

**Keywords:** Human behaviour, Computational science, Information technology

## Abstract

Identifying helpful information from large-scale online reviews has become a core issue in studies on harnessing wisdom-of-crowds. We investigated whether online reviews expressing dissenting opinions (i.e., outlier reviews) can provide helpful information. Using statistical and simulation methods with a large-scale dataset, we found that, compared with other online reviews, outlier reviews were deemed more helpful because they provided more sufficient, neutral, and concise information. To interpret these results, we considered that in collective behaviours, a prevalent social psychological process—conformity (i.e., changing one’s behaviour in response to pressure from others)—pressured reviewers expressing dissenting opinions. This motivated them to provide more convincing evidence (i.e., sufficient, neutral, and concise information). This study offers a simple yet effective approach for eliciting helpful information from many online reviews and deepens the understanding of the mechanism underlying collective online behaviour. Specifically, conformity was considered to cause biases in the collective behaviour of humans; however, this study revealed that conformity can elicit valuable outcomes in collective behaviour.

## Introduction

In recent decades, online reviews have become a valuable source of information for assisting people in making decisions on online platforms, such as whether to purchase a product^1^. Given the vast number of online reviews^2,3^, a prominent issue is how to effectively elicit helpful information for decision-making purposes^1–6^. In this regard, many previous studies^7–9^ have considered average aggregation to be a useful method. Specifically, these studies suggested that given a collection of different opinions in online reviews (e.g., different ratings for a collection of online reviews), the average opinion (e.g., the average rating for a collection of online reviews) can provide the most helpful information (e.g., the rating which best reflects the quality of a target product) compared with any individual opinion.

However, as recent studies have investigated online reviews in more detail, many^1,6,10^ have found that average aggregation is not a reliable way to elicit helpful information. The main reason for this is that opinions in online reviews are overwhelmingly positive^10^. For instance, on Amazon.com, a well-known online shopping platform, the average rating of all reviews is close to 4.2 out of 5^2^. More importantly, the average rating for different products on Amazon.com did not reflect the different features (e.g., quality) of these products^10^. Social psychological research and theory^11,12^ suggest that conformity—that is, changing one’s behaviour in response to pressure from others—can help explain these overwhelmingly positive ratings. When people express their opinions, they may conform to the majority opinion (i.e., the most frequently occurring opinion) in existing reviews. In other words, previously expressed opinions distort subsequent opinions to bring them closer to the majority opinion. Research has shown that one reason people conform is normative social influence, that is, people conform to avoid standing out negatively in others’ eyes or being viewed unfavourably^13,14^. Thus, regardless of a target product’s actual features (e.g., its quality), positive opinions such as 5-star ratings are more likely to become the majority opinion in online reviews. More specifically, conformity due to normative social influence tends to result in online reviews that follow a *J-shaped* distribution with overwhelming positivity. As a result, the average opinion (e.g., the average rating) only reflects the online reviews’ degree of overwhelming positivity and fails to offer helpful information.

**Figure 1:**
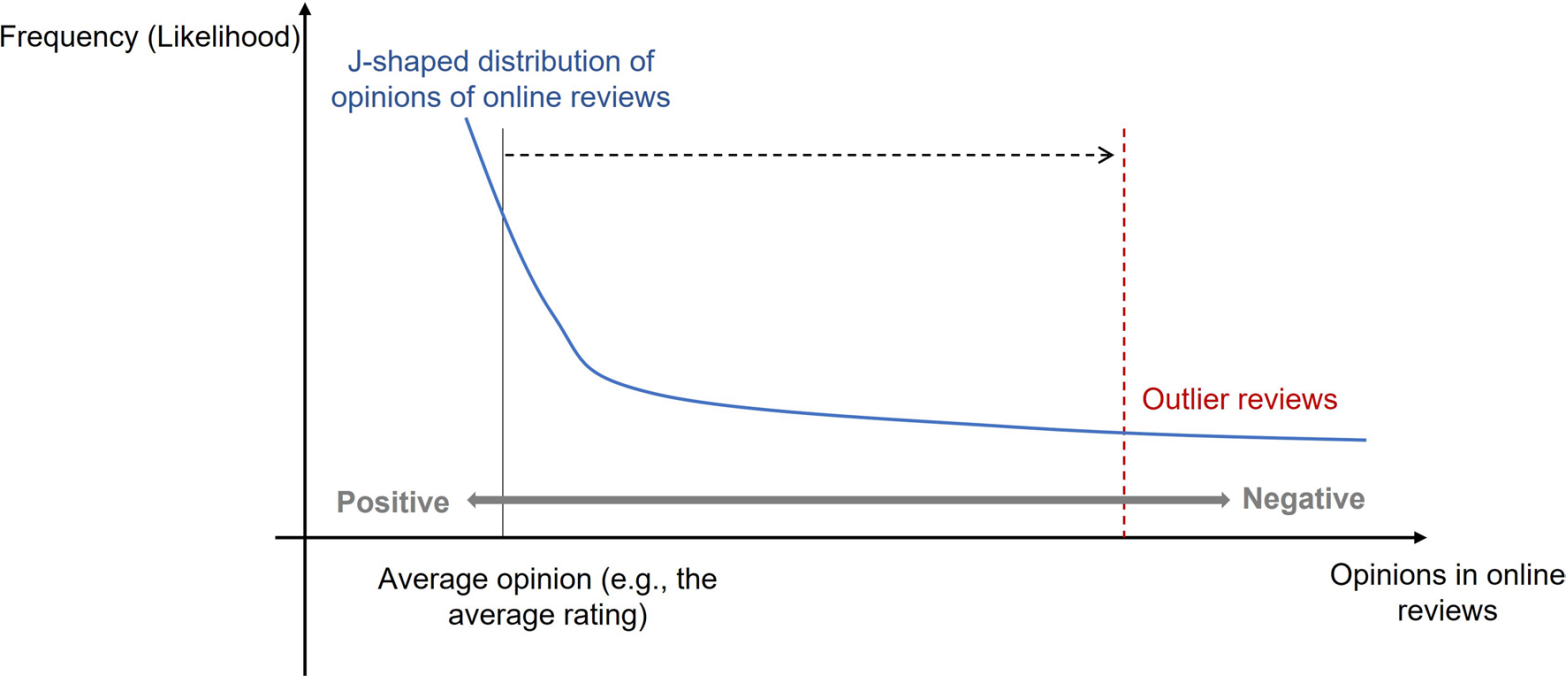
Illustration of the outlier reviews. The blue line shows a hypothetical but typical J-shaped distribution of opinions with overwhelming positivity in online reviews. The grey solid vertical line shows the average rating of the J-shaped distribution, and the red dotted vertical line illustrates the rating of typical examples of outlier reviews. The black dotted line reflects a large difference in rating between average and outlier opinions. Outlier opinions can be identified based on either a continuous or discontinuous distribution.

To find an alternative approach to elicit helpful information from online reviews, this research focused on outlier reviews. Outlier reviews refer to reviews with opinions which largely deviate from the average (i.e., the reviews with dissenting opinions; see Fig. 1). There is an interesting paradox in considering whether outlier reviews can be helpful for readers. From the perspective of wisdom-of-crowds research^7–9^, outlier reviews cannot be more helpful than other reviews, and conversely, these reviews may carry opinions with a larger bias because they often express minority opinions (i.e., opinions which are expressed by a small number of reviewers). However, social influence research in social psychology field^15–19^ suggests a different perspective: the minority opinion can influence the majority opinion by serving as a source of information about what is accurate or effective (i.e., informational social influence). Having strong accuracy goals has been shown to allow people to resist normative social influence, especially when they are prepared to explain their decisions^20^. Thus, if people who write outlier reviews are strongly accuracy-motivated, they can be expected to both resist normative positivity pressure and provide more compelling, less biased, and more helpful information.

Based on the above discussion regarding outlier reviews, in the following sections, we empirically investigate whether outlier online reviews can provide more helpful information for their readers’ decision-making and, if so, why. By analysing a large-scale dataset of online reviews, our research makes two contributions. First, it offers a simple but effective approach to eliciting helpful information from online reviews. While previous studies^1,4,7,21^ demonstrated various approaches for eliciting helpful information, these approaches require sophisticated analyses of each review’s content, while our study shows that based on simple analyses of reviews’ degrees of deviation from the average opinion (i.e., the outlier degrees of reviews), people can effectively identify helpful reviews. Thus, our research provides a less costly way to harness the online wisdom-of-crowds. More importantly, based on further investigation of the mechanism underlying as to why outlier reviews are helpful, this research deepens the understanding of the conformity and related social influence processes. These processes have always been considered to cause biases in the collective behaviours of humans on a mass scale^21–23^. However, our study reveals that they can also stimulate valuable outcomes in collective behaviour.

## Results

### Overview of the dataset and the metric of the outlier degree

To investigate the helpfulness of online outlier reviews, we employed data from a well-known online shopping platform, Amazon.com. By using an open dataset^24^ (see details in the subsection “General information regarding the Amazon.com review dataset” in the “Methods” section), we analysed approximately 53 million online reviews posted on Amazon.com (from May 1996 to October 2018). These reviews were related to more than nine million products belonging to seven major product categories on Amazon.com; the specific scale and categories of the dataset are shown in Table 1.

**Table 1:** Basic information of the review dataset.

Product category	Number of products	Number of reviews
Books	2,935,525	27,164,983
Clothing, shoes and jewellery	2,685,059	11,285,464
Electronics	786,868	6,739,590
Tools and home improvement	571,982	2,070,831
Cell phones and accessories	634,414	1,128,437
Automotive	932,019	1,711,519
Sports and outdoors	962,876	2,839,940
Sum	9,508,743	52,940,764

To identify outlier reviews in this large-scale dataset, we first measured each review’s degree of deviation from the average opinion (hereinafter, the outlier degree). The metric was computed based on five-star ratings for the reviews (i.e., a 5-point rating from five stars to one star). The outlier degree,$OD_{i}^{j}$, for review $i$ for product *j* was computed as follows:

$$OD_{i}^{j} = \frac{{|Rating_{i}^{j} - Mean\;(Rating_{k}^{j} )|}}{{St.\;dev\;(Rating_{k}^{j} )}}$$

where $Rating_{i}^{j}$ is the rating of review $i$, $Mean\;(Rating_{k}^{j} )$ is the average rating of all reviews of product $j$, and $St.\;dev\;(Rating_{k}^{j} )$ is the standard deviation of the ratings of all reviews for product $j$. This metric (hereinafter, the *absolute z-score method*) measured the extent to which review $i$’s rating deviated from the average rating of all reviews for the same product. The larger the $OD_{i}$, the larger the outlier degree of review $i$.

In addition, to examine the robustness of our results, we employed another method to calculate the outlier degrees. Previous research^25^ suggests that z-score can cause bias in detecting outliers when there are extreme values in datasets. Instead, it is more robust to use the median absolute deviation method (hereinafter, the *MAD* method; see section ‘S1’ of the ‘Supplementary Material’) to identify outliers. However, because ratings only comprise a narrow range of integer values—one to five on Amazon.com—we considered that there would be no extreme values, thus not largely distorting the outlier detection results. Therefore, we only used the MAD method as a supplementary approach, and the results are briefly reported in the “Results” section (see section ‘S1’ of the ‘Supplementary Material’ for further details).

As every review’s outlier degree was computed in comparison with other reviews for the same target product, we only used reviews where the target product received more than five reviews. Therefore, the 53 million English reviews analysed in this research were extracted from all 144,647,534 reviews for the products belonging to the seven categories under study. As previous research^26^ pointed out that the contents and rating distributions were different in different product categories, the reviews in different categories were analysed independently.

### Outlier reviews received more helpful votes

Using the above review dataset, we first investigated whether reviews with larger outlier degrees were considered more helpful to their readers. To measure the helpfulness of each review, we employed the number of votes metric for each review. The vote is a function provided by Amazon.com which allows users (i.e., the readers of reviews) to vote for the reviews which they consider helpful. Based on survey data, previous studies^4,26^ in management pointed out that reviews which received a very large number of votes can effectively help their readers make purchasing decisions. Therefore, in this research, we considered a review which received a very large number of votes as a more helpful one.

**Figure 2:**
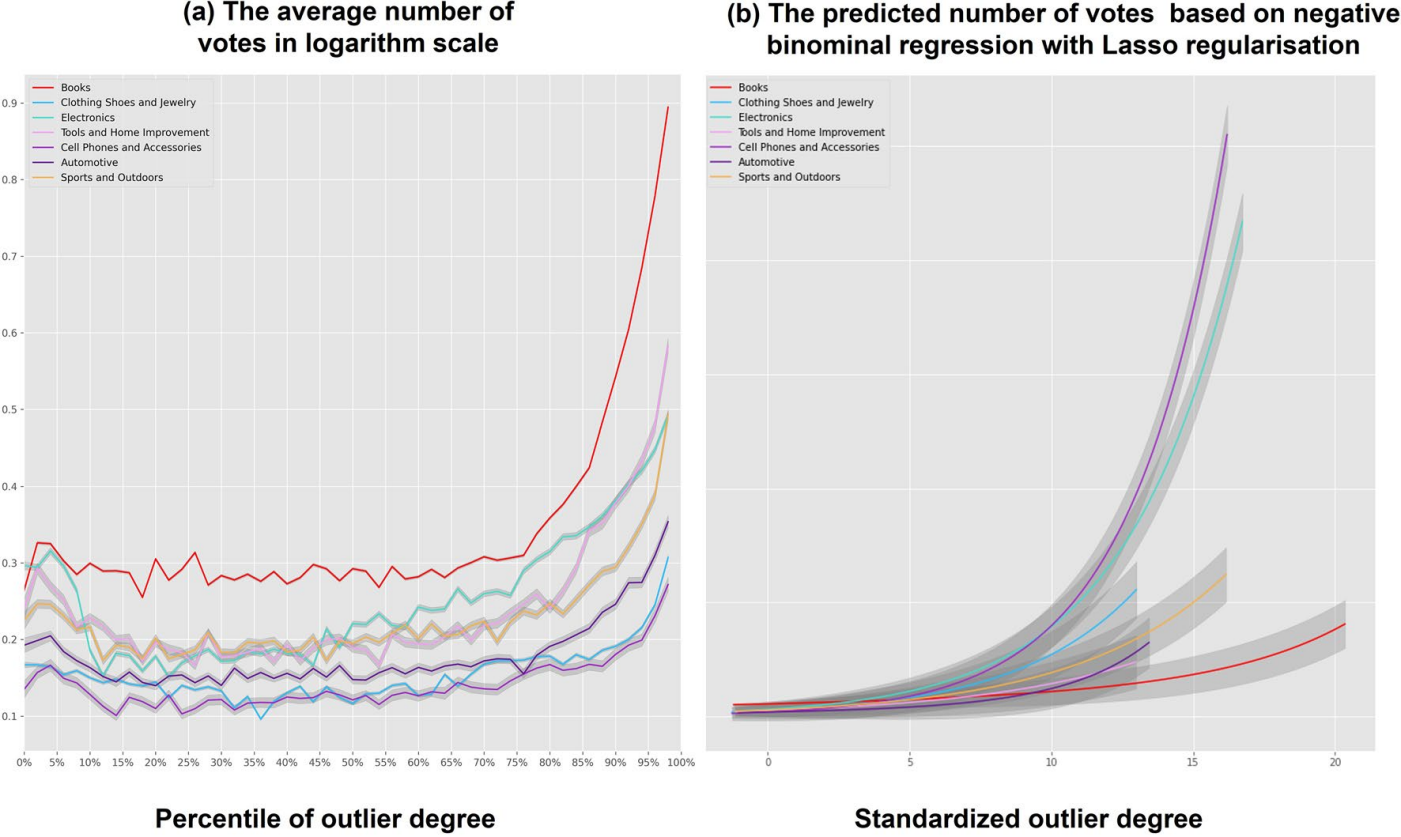
Illustration of the relationship between outlier degrees and the number of votes. (**a**) Shows the average number of votes for the reviews with different outlier degrees. The x-axis represents the percentile of the outlier degree for a review. The y-axis represents the average number of votes (in logarithm scale) for each review. The colours of lines reflect the results for reviews in different product categories. This figure shows that regardless of the different product categories, the reviews with large outlier degrees received far more votes from their readers than the reviews with small outlier degrees. (**b**) Shows the predicted number of votes for reviews with different percentiles of outlier degrees. The x-axis represents the value of the standardized outlier degree for a review. The y-axis represents the predicted number of votes (in logarithm scale) of each review using the negative binomial regression model. The lines show the predicted numbers of votes for reviews with different outlier degrees but with the same values (which are equal to the means) of the control variables. As in (**a**), the different colours of the lines reflect the results based on the regression models for the reviews in different product categories. (**b**) Shows that even when controlling all related variables, the reviews with large outlier degrees still received more favourable votes than the reviews with small outlier degrees across all seven different product categories.

Using this metric, we first investigated the relationship between a review’s outlier degree and the number of votes. The lines in Fig. 2a show the average number of votes for reviews with different outlier degrees. The colours of the lines reflect the results of reviews in different product categories. From Fig. 2a, we found that regardless of the different product categories, the reviews with large outlier degrees received far more votes than those with small outlier degrees. The results were consistent when using the MAD method to identify outlier reviews (see the details in Fig. S1).

However, based on these results alone, it is doubtful that outlier reviews were actually more helpful, and many related variables needed to be controlled further. Based on negative binomial regression models, we re-examined the relationship between the outlier degrees and the number of votes by controlling the following three groups of related variables (see the computation details of these control variables in the subsection “Regression models of the number of votes for reviews with different outlier degrees” in the “Methods” section; see the statistical information of these variables and their correlations in Table S1 and Figs. S1–S7 in the ‘Supplementary Material’):

- As contended in previous research^10^, the J-shaped distribution of review opinions could lead to a case where most reviews with smaller outlier degrees express particularly positive opinions, but some reviews with larger outlier degrees express negative opinions (e.g., those which rated the target product as one star; see the details of the distributions of positivity of reviews in S2 section in the ‘Supplementary Material’). Therefore, an alternative explanation for the results in Fig. 2a may be that reviews with negative opinions can receive a greater number of votes. To rule out this alternative explanation, the regression models first controlled the positive opinion expressed by the review based on two variables: (i) the rating positivity represented the degree of positivity or negativity expressed by the rating for the review (e.g., five stars or one star) and (ii) the content positivity represented the sentiment positivity for the content of the review.
- The number of votes for a review can highly correlate with the chance of the review being read by other users (i.e., its readers). Specifically, a review with a greater chance of being read can naturally receive more votes. To control the chance of being read, we added a rank in the review’s display (i.e., the rank at which the review appeared in the overall reviews for the same target product), as well as the length of the review, and then uploaded a timestamp for the review in our regression model. Based on previous research^4,26^, we believed that a review with a high rank in the display (e.g., the first to appear in the overall reviews), of a longer length, and that was uploaded earlier had a greater chance of being read by other Amazon.com users.
- Since the outlier degree was computed based on the distribution of the ratings for the same target product, this distribution may largely affect the relationship between the number of votes and the outlier degree^5^. For instance, if all reviews except one outlier review rated the target product as five stars, this outlier review may receive more attention from its readers than other outlier reviews with a flat distribution of ratings. To control for the influence of rating distribution for the target product, we added the mean and standard deviation of its ratings, as well as the number of reviews received for the target product as control variables in our regression model.

Figures S5–S11 in the ‘Supplementary Materials’ show that the correlations between control and independent variables were fairly high. Therefore, we further used Lasso regularisation to prevent the multicollinearity issue^27^. In Lasso regularisation, a regression model attempts to find the smallest model using the fewest variables to fit the dependent variables. Thus, when a variable has collinearity with other variables, the regression coefficients thereof will be set to zero. Only variables that independently affect the dependent variable have non-zero regression coefficients. In this sense, the LASSO regularisation effectively prevents multicollinearity among variables^27^. Therefore, a negative binomial regression model with LASSO regularisation was built.

In addition, we built a hierarchical regression model to further control the influence of different reviewers and periods (e.g., to control the cases where the reviews posted by a reviewer with more expertise, or posted close to *Black Friday*, always receive more votes; see the “Regression models of the number of votes for reviews with different outlier degrees” subsection in the “Methods” section for details). Specifically, in addition to the above control variables, the reviewer’s ID and the reviews’ post-dates were added to this supplementary regression model as fixed effects. The results of these supplementary models are consistent with those of the negative binomial regression model. As previous research^28,29^ in statistics pointed out that the values of the coefficients estimated by a hierarchical regression were less robust, the results of the hierarchical models are reported in section S3 in the ‘Supplementary Material’.

Based on the above regression models, we obtained the results shown in Fig. 2b, which represent the predicted number of votes for reviews with different outlier degrees (see the specific regression coefficients in Tables S2–S5 in the ‘Supplementary Material’). The results showed that even when all the above related variables were controlled, the reviews with large outlier degrees still received a greater number of votes than those with small outlier degrees across all the seven different product categories. We also built similar negative binomial regression and hierarchical models where the outlier degree was replaced by the independent variable based on the MAD method. The regression results of these models were consistent with the above model (see section ‘S3’ in the ‘Supplementary Material’).

From the results shown in Fig. 2, we concluded that outlier reviews were considered more helpful for their readers. An issue that remained was what made outlier reviews more helpful. In the next subsection, we investigated this issue further.

### Outlier reviews provided information of a higher quality

A recent study^30^ found that in collective behaviour, people (e.g., a Wikipedia editor) who expressed dissenting opinions provided more high-quality information to support their own opinions than those who expressed opinions similar to the majority one. This result can be interpreted as follows: because of the conformity and related social influence processes, people expressing dissenting opinions are under significant peer pressure; thus, they are compelled to reinforce their own opinions by providing evidence with high-quality information. Based on this previous study, we hypothesised that reviewers who submitted outlier reviews may face a similar situation: since the outlier reviews violate the conformity and related social influence processes, the reviewers may be forced to provide evidence with higher quality information to persuade their readers.

To investigate why outlier reviews were more helpful for their readers, we then analysed the quality of the information contained in the written content (i.e., texts) of the reviews according to the different outlier degrees. Although information quality is a complex concept that includes over 10 different dimensions^31,32^, there are only three dimensions that mainly affect the helpfulness of a review: sufficiency, subjectivity, and conciseness^32^. Sufficiency indicates the extent to which a review offers sufficient evidence to explain its opinions. This can be effectively measured by the information entropy of the text contained in the review^33,34^ (see the computational details in the subsection “Metrics of information quality of review contents” in the “Methods” section). Higher information entropy indicates higher sufficiency of the review. Subjectivity indicates the extent to which the evidence provided by a review is based on subjective feelings rather than objective facts. As subjective feelings do not provide helpful information to assist the review’s readers when making decisions, helpful reviews require low subjectivity. We measured subjectivity using a sentiment analysis of the text contained in the reviews (see the computational details in the subsection “Metrics of information quality of review contents” in the “Methods” section). Finally, conciseness indicates the extent to which the information in a review is expressed concisely and clearly. If the conciseness of a review is low, it is difficult for readers to elicit information from this review, even if the sufficiency and subjectivity of the review are perfect. Therefore, helpful reviews require high conciseness. According to previous research^31,32^, we computed the metric of conciseness as the ratio between the information entropy of a review and the length of this review (see the computational details in the subsection “Metrics of information quality of review contents” in the “Methods” section). A larger value of this ratio reflects higher conciseness of a review.

**Figure 3:**
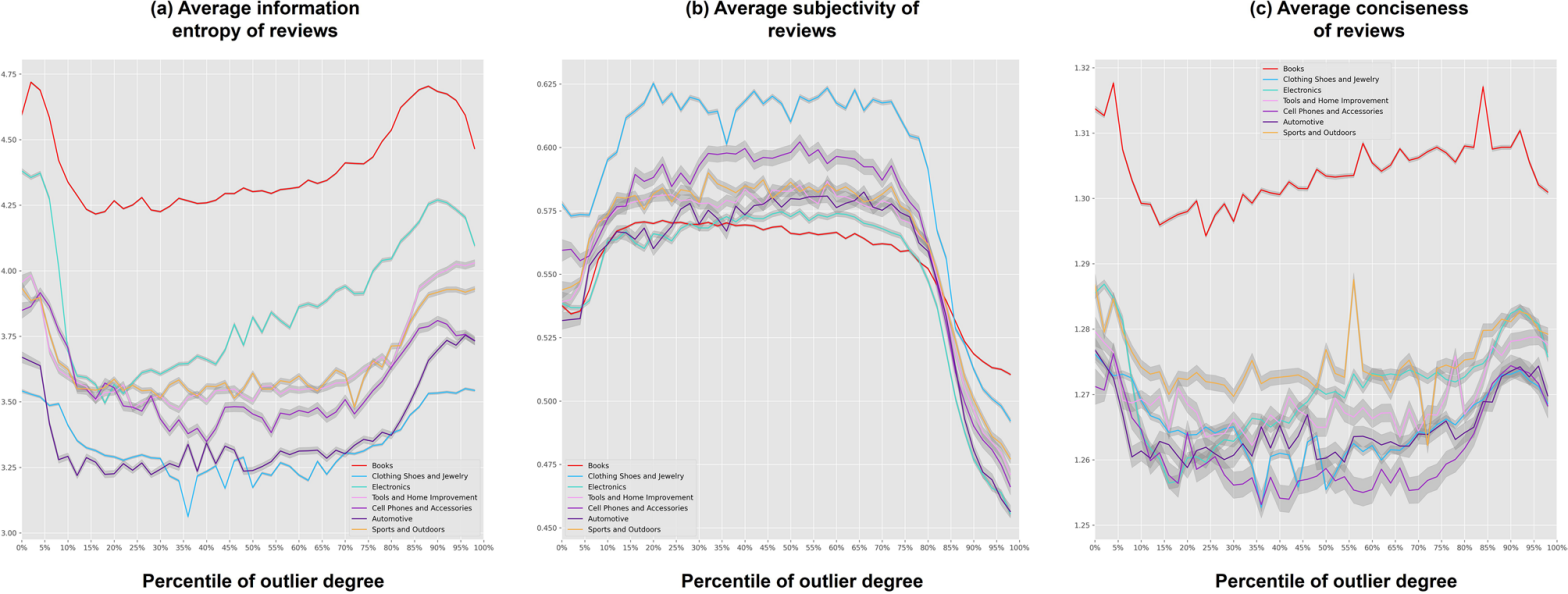
Illustration of the relationship between outlier degrees and information quality. In (**a**–**c**), the x-axis indicates the percentile of values of outlier degrees. The y-axis indicates the average information entropy of the text contained in each review in (**a**), average subjectivity of the text contained in each review in (**b**), and the average conciseness of each review in (**c**). The lines show the different information entropies of the reviews with different outlier degrees in (**a**), the different subjectivities of the reviews with different outlier degrees in (**b**), and the different conciseness of the reviews with different outlier degrees in (**c**). The different colours of the lines reflect the results based on the regression models for the reviews in different product categories. In summary, this figure shows that compared to other reviews, the outlier reviews provided information with smaller subjectivity, larger information entropy and higher conciseness.

Using the above three metrics, Fig. 3 shows the relationship between the information quality and outlier degree of the reviews. In Fig. 3a, we found that the relationship between outlier degree and information entropy was U-shaped. In Fig. 3c, the same U-shaped relationship was also found between outlier degree and conciseness. These relationships indicate that the information entropies and conciseness of reviews with very high outlier degrees were equivalent to those of reviews with very low outlier degrees, both of which were higher than those of reviews with medium outlier degrees. It is also worth noting that the U-shaped relationships implied that when considering the efforts for writing a review, the reviewers seemingly exerted equal efforts to write either a normal (i.e., small outlier degree) or an outlier review. Therefore, factors such as lack of time and laziness could hardly be considered as the cause of few votes for normal reviews. Nonetheless, more importantly, Fig. 3b shows that the reviews with higher outlier degrees provided less subjective text (i.e., lower subjectivity). All these results were consistent across the reviews for different product categories (shown by differently coloured lines in Fig. 3). These results were also consistent when using the MAD method to identify outlier reviews (see Figs. S2–S4). To summarise, these results reflected that the outlier reviews provided information with high sufficiency, low subjectivity, and high conciseness. Therefore, by taking the three aspects of information quality into account, we can conclude that the outlier reviews provided information of a higher quality than other reviews.

When taken together, the results in the above two subsections indicate that (1) the outlier reviews were considered more helpful for their readers than other reviews and (2) the outlier reviews provided information of a higher quality than other reviews. These results imply that high-quality information may correlate with the high helpfulness of outlier reviews. Following these results, we employed simulation methods to further examine whether there is a correlation between the greater number of votes for outlier reviews and their high information quality in the next section.

### Simulation of the relationship between information quality and the number of votes for outlier reviews

To examine whether the greater number of votes for outlier reviews correlated with their high information quality, we employed two simulation models. These two simulation models consisted of a collection of hypothetical readers and candidate reviews. Each hypothetical reader decided whether they would vote for a candidate review in the models. The difference between the two models is that in one model (hereinafter, the *full model*), the hypothetical reader decided whether to vote for a candidate review considering candidate review’s information quality (i.e., sufficiency, subjectivity, and conciseness). In the other model (hereinafter, the *Bayesian learning model*), the reader decided whether to vote *without* considering the information quality of the candidate review. By comparing how close the distribution of the votes generated by each simulation model was to the distribution of the votes in the real data, we examined whether readers in the real world voted for the outlier reviews by considering the outlier reviews’ information quality. As a result of comparing to the Bayesian learning model, the distribution generated by the full model was found to be significantly closer to the distribution of votes in the real world; this result indicates that the readers in the real world decided whether they would vote for a review considering its information quality. Otherwise, the result indicates that the information quality of each review did not affect the readers’ decisions of voting.

We will explain our simulation models more specifically; in the full model, a hypothetical reader decided whether they would vote for a candidate review considering its information quality. The readers could not observe the information quality of *every* candidate review but only of those they chose to read. As a result, two steps were designed to determine whether a reader would vote for the reviews^35,36^ (see the details of these two steps in subsection “Mathematical details of the two steps in the simulation models” in the “Methods” section): in the first step (hereinafter, the *pick-up step*), a reader would choose a small number of reviews (e.g., 10 reviews) to read from many candidate reviews (e.g., 100 reviews). To determine which reviews would be chosen by the reader, based on previous studies^35,36^, we hypothesised that (1) the readers voted one by one and (2) the *n*th reader would choose the reviews referring to the votes held by the *n* − 1 previous readers and the age of the reviews (i.e., the number of days a review has been posted). In the second step (hereinafter, the *voting step*), the reader would know how many pieces of high-quality information were contained in the reviews which they chose. The amount of high-quality information was computed according to the subjectiveness and the sufficiency of each review. The reader then decided whether to vote for each review by comparing the number of pieces of high-quality information contained in the review with their own threshold. For instance, a review which contained *m* pieces of high-quality information was chosen by reader *n*. If *m* was larger than the threshold of reader* n*, this review would be *possible* to receive a vote from reader *n*. The probability for this review to receive a vote from reader *n* is further decided by the conciseness of this review and the number of votes which have been received by this review. In a different case, if *m* was less than the threshold of reader *n*, the review would *also* have a chance to receive a vote from reader *n*. This chance is decided by the number of votes which have been received by this review.

Different from the full model, the hypothetical readers in the Bayesian learning model vote for the candidate reviews *without* considering the information quality (i.e., sufficiency, subjectivity, and conciseness) of each review. Specifically, the readers in the Bayesian learning model followed the same pick-up step as those in the full model. However, in the voting step, the readers in the Bayesian learning model voted for the reviews *only* based on the number of votes which these reviews have received and ignored the information quality of these reviews.

The pick-up step and the voting step were designed to bring our two simulation models closer to real-world situations where the vote process is affected by the selective attention and social influence mechanisms^35,36^. The selective attention mechanism indicates that readers in the real world have to decide which reviews to vote for in a situation where they can only read a small number of the reviews^37^. Under this situation, the readers will tend to select the reviews which have already received more votes and have been posted recently^35,36^. Since a review which has not been read cannot receive a vote, the selective attention mechanism is one of the keys which shape the distribution of the number of votes. A previous study^35^ also pointed out that even if a review with high-quality information was read by a reader, the review could probably fail to receive a vote from the reader. Two reasons may explain this. First, the reader may fail to elicit the high-quality information from the review because the review is difficult to understand. Based on previous studies^31,32^, the ease of understanding of a review was highly correlated with the conciseness of the review. Therefore, if a review includes a large amount of high-quality information but has a low conciseness, there is a chance for it to lose the vote from the reader. Second, even if a reader successfully elicits the high-quality information, the social influence mechanism could hinder this reader to vote for the review. It was found that if a reader finds that there is no other reader voting for a helpful review, they may lose the motivation to vote for the review^35^. In addition, the social influence mechanism also allows a reader to vote for a review which only includes low-quality information but has already received many votes^35,36^. Through the two steps, our simulation models took all above possible cases into account. Therefore, by comparing the results from the full model and Bayesian learning model, we can investigate how the information quality of reviews affects the distribution of votes under the influences of the selective attention and social influence mechanisms during the voting process.

Table 2 shows the results of the two models. The number of readers was set to 10,000, and the number of candidate reviews was set to 100 in both two models. All other parameters in the two models were set to represent the voting behaviours in the real world based on previous research^35^ (see the details in subsection “Mathematical details of the two steps in the simulation models” in the “Methods” section). Considering the random fluctuations, we ran simulations for each model 50 times and computed the distribution of votes based on the mean of the results of each simulation. To measure how close the distribution of votes generated by each of the two models was to the distribution in the real data, we measured the distance between the distribution of votes in the real data and that generated by each of the simulation models by employing the cross-correlation coefficient^38^ and the *Kullback–Leibler divergence* (hereinafter, *KLD*)^39^ between the distributions. A larger value of the cross-correlation coefficient and a smaller value of the KLD indicate that the distribution generated by the simulation model was closer to the distribution in the real data. Additionally, considering that the Bayesian learning model included fewer parameters than the full model (i.e., four parameters *vs* five parameters; see the details in the “Mathematical details of the comparisons of the simulation models” in the “Methods” section), we also employed *Akaike information criterion* (hereinafter, *AIC*)^40^ to examine whether the distribution generated by the full model was closer to the distribution in the real data by considering that the full model used more parameters. A smaller value of the AIC indicates that the distribution generated by the simulation model was closer to the distribution in the real data. The values of all these metrics were reported in Table 2 and the computations of these metrics can be found in “Mathematical details of the comparisons of the simulation models” in the “Methods” section. To summarise the results in Table 2, all results indicate that regardless of the data in different product categories, the distributions of votes generated by the full model were significantly closer to that in the real data (i.e., with larger cross-correlation coefficients, smaller KLDs, and smaller AICs) than those generated by the Bayesian learning model. Finally, to investigate the impact of the ratio between the number of the hypothetical readers and the number of candidate reviews on the result, we ran additional simulations by setting the number of readers to 5000 and 20,000. All additional simulation results were consistent with the results shown in Table 2. The specific cross-correlation coefficients are reported in Table S4. These results indicate that the distribution of the reviews’ votes was largely affected by the information quality of reviews.

**Table 2:** Cross-correlation coefficients, Kullback–Leibler divergences (KLDs), and Akaike information criterions (AICs) resulting from different simulation models.

Product category	Full model			Bayesian learning model		
	Cross-correlation coefficient	KLD	AIC	Cross-correlation coefficient	KLD	AIC
Books	0.83***	0.11	1165.58	− 0.16	0.54	1287.52
Clothing shoes and jewellery	0.59***	0.18	1166.02	0.03	0.42	1275.43
Electronics	0.14**	0.11	809.42	− 0.32***	0.55	1298.01
Tools and home improvement	0.34***	0.22	1055.68	− 0.07	0.47	1273.06
Cell phones and accessories	0.10*	0.31	1141.10	− 0.21*	0.49	1286.68
Automotive	0.50***	0.23	1213.46	0.09	0.42	1269.52
Sports and outdoors	0.51***	0.22	1111.59	0.03	0.44	1276.65

*p < 0.1, **p < 0.05, ***p < 0.01.

## Discussion

All the results obtained indicate that, compared to other online reviews, outlier reviews are more helpful for their readers. Based on content analyses and simulations, the results implied that the helpfulness of outlier reviews was highly correlated with the more sufficient, neutral, and concise information (i.e., high-quality information) provided by these reviews. To further interpret the results, we considered that the conformity due to normative social influence caused the high-quality information of outlier reviews^22,23,30^. Under this mechanism, the outlier reviewers faced a kind of peer pressure in that they were forced to provide more convincing evidence to support their own divergent opinions. As a result, this peer pressure made the outlier reviewers exert informational social influence and resulted in outlier reviews containing more helpful information about the target product.

This research makes two contributions. First, at an applied level, identifying helpful information has become a core issue in studies involving the harnessing of wisdom-of-crowds^1^. Many previous studies^1–4,21^ have proposed diverse methods to elicit helpful information from large-scale online reviews. However, these previous approaches always required sophisticated analyses for the content of reviews, which makes the elicitation of vital information costly. Our approach only requires limited statistical information on the outlier degrees of reviews to identify the helpful ones. Therefore, this study will provide future research a less costly technique to identify helpful reviews and to investigate further topics (e.g., how helpful reviews were generated). In practical terms, this approach can also help online platforms (e.g., Amazon.com) in the real world to harness the online wisdom-of-crowds more effectively. Instead of analysing all online reviews, focusing on outlier reviews can help these platforms to identify helpful information more effectively and affordably. More specifically, if Amazon.com can weigh the outlier degree in its review recommendation system, it may help the users to decide more easily. Second, at the theoretical level, by investigating why outlier reviews are more helpful, this study advances the understanding of how conformity and related social influence processes affect the collective behaviour of people on a mass scale. In contrast to previous studies^1,2,11–14^ which emphasised conformity and related social influence processes as the reason for biases in collective behaviour, this study implies that the peer pressure brought about by the conformity can also motivate people to produce helpful information during collective behaviour.

Despite these contributions, we must acknowledge three limitations of our study. First, this study only focused on reviews of products available at Amazon.com. Future research can further discuss whether the results are similar when focusing on the reviews for services instead of products. In that case, processes related to emotional states could also explain why outlier reviewers can yield helpful information, since previous research^1^ has pointed out that negative experiences may lead reviewers to express more detailed, less biased information. Negative emotional states were controlled in our analyses, and our results showed that this factor is not a main factor causing the greater number of votes received by outlier reviews. Future research can explore the effect of emotional state on outlier review content. Second, by comparing the full model to the Bayesian learning model, we have seen that the high information quality was a contributing factor to many votes for outlier reviews. However, our full simulation model can still be improved. The results of our full model in Table 2 implied that the information quality of reviews might not fully explain the distribution of votes on Amazon.com. Thus, future research can develop a more sophisticated simulation model for generating more accurate predictions. Third, through this research, we showed that focusing on outlier reviews is an effective way to elicit helpful information. However, normal reviews also contain immensely helpful information, although eliciting it is costly. Therefore, future studies should discuss how to effectively elicit helpful information from reviews.

Finally, future studies should explore the mechanism of generating outlier reviews. In addition to the high information quality of outlier reviews, Fig. 3 also shows that reviews with small outlier degrees (i.e., around 5 percentile) had higher information quality than those with a middle outlier degree (i.e., around 50 percentile). This is especially apparent considering their information entropy and conciseness. In section ‘S5’ of the ‘Supplementary Material’, we conducted supplementary analyses to show that this result might be caused by the difference between the reviewers’ experiences. Furthermore, we found that reviewers with fewer experiences had more opportunities to generate outlier reviews. These results were similar to the results of previous research on creative activities^41^, which found that people with less experience had more opportunities to generate high performance. In addition, the supplementary analyses in section ‘S6’ showed that more outlier reviews were posted from June to July yearly. By investigating what factors activated these occurrences, future studies can deepen the discussions of outlier reviews.

## Methods

### General information regarding the Amazon.com review dataset

In this study, we used an open dataset which included 52,940,764 online reviews posted between May 1996 and October 2018 in English on Amazon.com^24^. These reviews were selected from a total of 144,647,534 reviews worldwide on the condition that their target products received more than five reviews. The review data included (1) the target product under review, (2) the reviewer’s (i.e., author’s) ID, (3) the upload timestamp, (4) the rating, (5) the content text of the review, (6) the number of votes received, and (7) the rank at which the review appeared in the overall reviews for the same target product. Using each review’s rating, we computed the outlier degree (i.e., the degree of deviation from the average opinion; see details in the subsection “Overview of the dataset and the metric of the outlier degree” in the “Results” section) of every review. The usage of the other review features referred to above are detailed below.

### Regression models of the number of votes for reviews with different outlier degrees

To investigate whether the reviews with larger outlier degrees were considered more helpful for their readers, we employed the negative binomial regression in the subsection “Outlier reviews received more helpful votes” in the “Results” section. In these negative binomial regression models, the number of votes received by the target review was the dependent variable and the outlier degree was the independent variable. In addition to these two variables, the models included the following eight control variables:

- Rating positivity, which reflected the degree of positivity or negativity expressed by the review’s ratings, was represented by a categorical variable. When the rating of the target review was five or four, the value of the categorical variable was set to 1 which indicated a positive rating; when it was three, the value was set to 0 which indicated a neutral rating; and when it was two or one, the value was set to − 1 which indicated a negative rating^4,22,26^.
- Content positivity represented the sentiment positivity for the content of the target review. It was a variable with a value in the range of 1 to − 1 computed by TextBlob^42–44^, a widely used sentiment analysis module in Python. According to previous studies^42–45^, the TextBlob module uses a Naïve Bayes model that was trained on an online movie review dataset. Thus, this model can effectively elicit sentiment positivity, especially from short texts such as online reviews or tweets. Specifically, if the content positivity value is 1, this means that the text content of the target review is fully positive (i.e., praising); 0 means it is neutral; and − 1 means it is fully negative (i.e., criticising).
- The rank in the display of the target review represented the rank in which the review appeared in the overall reviews for the same target product. If this variable was 0, the target review was the first to appear in the overall reviews and if it was 99, it was the 100th review to appear in the overall reviews.
- The length of the target review was represented by the number of words in the content text of the target review. In the regression models, the logarithm scale length was used.
- The upload timestamp of the target review represented when the target review was uploaded. A smaller value indicated an earlier upload.
- The mean of the target product’s star rating.
- The standard deviation of the target product’s star rating.
- The number of reviews received by the target product.

The statistical information for these variables and the correlations among them can be found in section S3 of the ‘Supplementary Material’. As explained in the “Results” section, the correlations between control variables were fairly high. Therefore, we used Lasso regularisation to prevent multicollinearity. In Lasso regularisation, lambda is the penalty parameter. As lambda increases, the model becomes stricter towards unnecessary variables; that is, more variables will be removed from the model (i.e., the coefficients will be set to zero). The best lambdas reported in the ‘Supplementary Material’ were estimated using the AIC^27^.

In addition, we built a hierarchical regression model to further control the influences of different reviewers and dates. This supplementary regression model controlled reviewer IDs and the review post-dates as fixed effects. That is, the hierarchical regressions controlled the cases where reviews posted by reviewers with more expertise or posted close to Black Friday can receive more votes. As previous research in the field of statistics^28,29^ pointed out that estimating fixed effect may make the estimation of coefficients’ values of the negative binomial regression model less robust, we used a linear model (i.e., ordinary least squares) instead of the negative binomial regression model to control for the influence of reviewers. The linear model shared the same dependent variable (i.e., the number of votes; but on the logarithmic scale in this model), the same independent variable (i.e., the outlier degree), and the same control variables with the negative binomial model. The only difference was that the linear model estimated a different intercept for each reviewer. The specific regression coefficients of the negative binomial model and of the linear model can be found in section S2 of the ‘Supplementary Material’. The results were consistent with each other.

In both the negative binomial and hierarchical regression models, the independent (i.e., outlier degrees) and control variables were standardised.

### Metrics of information quality of review contents

According to previous studies^31,32^, we measured the information quality of a target review in terms of sufficiency, subjectivity, and conciseness.

The *information entropy* of the text contained in a target review was used to represent its sufficiency^31,32^. The computation of the information entropy was as follows:

$$H_{i} = - \mathop \sum \limits_{w \in i} p_{w} \cdot \log_{2} p_{w}$$

where *w* indicates the words which appeared in the target review *i* and $p_{w}$ indicates the ratio of word *w*’s occurrence in review *i*. This metric reflects the minimum cost for transmitting a review when the review was encoded as binary digits. According to information theory, this transmitting cost measures the number of bits of information contained in the review^31,32^. Therefore, higher information entropy indicates higher sufficiency of the review.

Subjectivity was measured by a sentiment analysis module in Python: TextBlob. Subjectivity is a variable with a value ranging from 0 to 1. If the value is 0, this means that the evidence provided by a review text is fully objective; in contrast, if it is 1, the evidence provided by the review text is fully subjective. As is the case for the computation of content polarity (i.e., a traditional sentiment analysis approach), which uses the text contained in the target review as an input and a Naïve Bayes model that was trained on an online movie review dataset and is known to be an efficient way of estimating the subjectivity of online reviews^42–45^.

Finally, conciseness was measured by the ratio between the information entropy of the target review and the length (i.e., the number of words) of the target review^31,32^. This ratio reflects averagely how many bits of information is expressed by one word in the target review. A large value of this ratio indicates a high conciseness of the target review.

### Mathematical details of the two steps in the simulation models

As discussed in the “Results” section, we employed two simulation models (the full model and the Bayesian learning model) to show how high-quality information contained in outlier reviews attracted a greater number of votes. In these simulation models, a collection of readers voted for a collection of candidate reviews one by one. Two steps were designed to determine whether a reader would vote for a candidate review or not: the pick-up step and voting step. Each candidate review had different levels of sufficiency, subjectivity, and conciseness. These values were set according to the average sufficiency, subjectivity, and conciseness calculated for the real reviews with the same percentiles of outlier degrees. For example, the sufficiency of the 20th review in 100 hypothetical reviews equalled the average information entropy of the real reviews with the bottom 20% outlier degrees.

In the pick-up step of the full model, the *n*th reader decided which *k* reviews (e.g., 10 reviews) they were going to read, referring to the votes held by the *n* – 1 previous readers and the age of the reviews. It was found that in the real world, *k* follows a Gaussian distribution with a mean equal to 7.4 and a standard deviation as 2.4^35^. Therefore, for each reader, their *k* was randomly sampled from above a normal distribution, that is, $k \sim N\;(7.4, \;2.4)$. Then, following a previous study^35^, the probability for each review being selected was computed as follows:

$$pr_{i}^{R} = \frac{1}{{1 + e^{{\beta_{0} + \beta_{1} V_{i} + \beta_{2} R_{i} + \beta_{3} Age_{i} }} }}$$

where $pr_{i}^{R}$ is the probability of selecting review *i* to read; $V_{i}$ represents the number of votes for the review *i* received from the previous *n* − 1 readers, and $R_{i}$ represents the number of previous readers who had chosen to read the review *i*. $Age_{i}$ represents the number of days from the date when the review *i* was posted to the date when the dataset was collected (31st October 2018). $\beta_{i}$s are the parameters which decide how $V_{i}$, $R_{i}$, and $Age_{i}$ affect the probability of review *i* being read. To infer the values of these parameters, a previous study^35^ conducted an experiment to collect a dataset recording the real pick-up processes of 64 undergraduate students. Based on this dataset, the values of $\beta_{i}$s were estimated (i.e., $\beta_{0} = 1.599, \;\beta_{1} = - 0.568, \;\beta_{2} = 0.170, \;\beta_{3} = 0.100$). In this research, we set $\beta_{i}$s to these values^35^. Additionally, all mathematical details in the pick-up step of the Bayesian learning model were the same as those of the full model.

After choosing the reviews in the voting step of the full model, every reader decided whether to vote for the reviews that they had chosen, referring to the sufficiency, subjectivity, and conciseness of each review. First, based on sufficiency (i.e., information entropy, $H_{i}$) and subjectivity, the number of pieces of high-quality information (hereinafter, *HQIn*) of a review was computed as follows:

$$HQIn_{i} = H_{i} \cdot (1 - Subjectivity_{i} )$$

This indicator refers to how many pieces of neutral information (i.e., high-quality information) a review provides.

For reader *n*, a threshold $h_{n}$ represents the number of pieces of $HQIn$ which was expected to get from the reviews which reader *n* has read. According to previous research^46^, the threshold $h_{n}$ can be interpreted as reflecting the risk preference of reader *n* during their decision-making: a risk-averse reader requires more information for decision-making than a risk-seeking reader. Therefore, the threshold $h_{n}$ was sampled from a Gaussian distribution which represents risk preferences among the entire readerships. In the simulations, the mean of this Gaussian distribution was set to the average value of the *HQIn*s of all reviews, and the standard deviation was set to the standard deviation of all reviews’ *HQIn*s. When the $HQIn$ of review *i* is equal to or larger than $h_{n}$ pieces, the review *i* provides enough *HQIn* to reader *n*. However, even if the review *i* includes enough *HQIn*, the reader *n* cannot always understand the review *i* (i.e., elicit *HQIn*)^35,36^. Based on previous research^31,32^, the probability for reader *n* to successfully understand the review *i* (i.e., $pr_{i}^{U}$) was decided by the conciseness of review *i*. Additionally, based on experimental data, previous research^35^ found that considering all reviews on Amazon.com, for 15% reviews, the $pr_{i}^{U}$ was equal to 0.9; for 40% reviews, $pr_{i}^{U}$ was equal to 0.7; for 45% reviews, $pr_{i}^{U}$ was equal to 0.5. Following on these previous studies^31,32,35^, the $pr_{i}^{U}$ of each review in our simulation models was set as follows: for the reviews with the top 15% conciseness, the $pr_{i}^{U}$ was averagely equal to 0.9; for the reviews with conciseness at the percentile from 15 to 55%, the $pr_{i}^{U}$ was averagely equal to 0.7; for the reviews with the bottom 45% conciseness, the $pr_{i}^{U}$ was averagely equal to 0.5.

In addition to $HQIn_{i}$ and $pr_{i}^{U}$, the previous votes (i.e., $V_{i}$, $R_{i}$) also affected the voting process of reader *n*. The social influence mechanism indicates even if the review *i* provides enough *HQIn* and the reader *n* successfully elicits it, the reader *n* may still choose not to vote for the review *i*. If there is no other reader voting for a helpful review, the reader *i* may also lose the motivation of voting for the review^35^. In contrast, a reader may also vote for a review which has already received many votes even if they failed to elicit enough high-quality information from this review^35,36^. To combine the influence from the information quality of review *i* (i.e., $IQIn_{i}$ and $pr_{i}^{U}$) and the previous votes (i.e., $V_{i}$, $R_{i}$), the reader *n* was considered as a Bayesian learner. To be more specific, this means that (1) without any information about the information quality and the previous votes of review *i*, the reader *n* will randomly (i.e., with a probability equal to 0.5) vote for the reviews read by them; (2) when they got the information about the information quality and the previous votes of review *i*, this information would be used to update the probability to vote for the review *i* (i.e., $pr_{i}^{(n)}$) based on Bayesian rules as follows:

$$pr_{i}^{(n)} \sim Beta\left( {1 + \alpha_{i}^{(n)} ,1 + \beta_{i}^{(n)} } \right)$$

$$\alpha_{i}^{(n)} = W_{n} \cdot {\mathbf{1}}\left( {HQIn_{i} } \right) \cdot pr_{i}^{U} + V_{i}$$

$$\beta_{i}^{(n)} = W_{n} \cdot \left( {1 - {\mathbf{1}}\left( {HQIn_{i} } \right) \cdot pr_{i}^{U} } \right) + R_{i} - V_{i}$$

$${\mathbf{1}}\;\left( {HQIn_{i} } \right) = \left\{ {\begin{array}{*{20}l} {0;} & {\quad if\;HQIn_{i} \ge h_{n} } \\ {1;} & {\quad if\; HQIn_{i} < h_{n} } \\ \end{array} } \right.$$

where $Beta\left( {1 + \alpha_{i}^{(n)} ,\;1 + \beta_{i}^{(n)} } \right)$ is the posterior distribution of the probability for reader *n* to vote for the review *i*, and ${\mathbf{1}}\left( {HQIn_{i} } \right)$ is the indicator function to show whether $HQIn_{i}$ is larger or equal to $h_{n}$ or not. $W_{n}$, as a parameter, reflects the extent to which the vote was decided based on the reader *n*’s own assessment of the information quality of review *i.* Further, if $W_{n}$ is equal to zero, it means that the vote was decided only based on the previous votes ($V_{i}$, $R_{i}$)*.* To the best of our knowledge, there were no previous studies which explored the value of $W_{n}$ in the voting process in the real world. Therefore, this parameter was optimised in the full model by minimising the KLD of the model (see the computation details of KLD in the next section).

### Mathematical details of the comparisons of the simulation models

To compare between the two simulation models how close the distribution of votes generated by each of the two models was to the distribution in the real data, we measured the distance between the distribution of votes in the real data and that generated by each of the simulation model by employing the cross-correlation coefficient, the KLD), and the AIC^38–40^. The cross-correlation coefficient was computed as follows^38^:

$$\rho = \frac{{E\left[ {\left( {V_{i}^{real} - E\left[ {V_{i}^{real} } \right]} \right)\left( {\widehat{{V_{i} }} - E\left[ {\widehat{{V_{i} }}} \right]} \right)} \right]}}{{\sqrt {E\left[ {\left( {V_{i}^{real} - E\left[ {V_{i}^{real} } \right]} \right)^{2} } \right]} \sqrt {E\left[ {\left( {\widehat{{V_{i} }} - E\left[ {\widehat{{V_{i} }}} \right]} \right)^{2} } \right]} }}$$

where $V_{i}^{real}$ is the number of votes which was received by reviews with outlier degree at percentile *i* in the real data, and $\widehat{{V_{i} }}$ is the number of votes which was received by reviews with outlier degree at percentile *i* generated by the simulation models. This metric reflects whether the number of votes in the real world and the number of votes generated by the simulation models are linearly correlated or not. A larger value of the cross-correlation coefficient indicates that the distribution generated by the simulation model was closer to the distribution in the real data.

KLD reflects the distance between the distribution generated by the simulation model and the distribution in the real data. It was computed as follows^39^:

$$KLD(D^{real} ||\hat{D}) = \mathop \sum \limits_{i} P^{real} (i)\log \left( {\frac{{P^{real} (i)}}{{\hat{P}(i)}}} \right)$$

$$P^{real} (i) = \frac{{V_{i}^{real} }}{{\mathop \sum \nolimits_{i} V_{i}^{real} }}$$

$$\hat{P}\left( i \right) = \frac{{\widehat{{V_{i} }}}}{{\mathop \sum \nolimits_{i} \widehat{{V_{i} }}}}$$

A smaller value of the KLD indicates that the distribution generated by the simulation model was closer to the distribution in the real data.

The AIC also reflects the distance between the distribution generated by the simulation model and that in the real data. Additionally, it considers the different numbers of parameters used in the two models. In other words, by using AIC, we examined whether the distribution generated by the simulation model could be considered as closer to the distribution in the real data, considering that the full model used more parameters than the Bayesian learning model. It was computed as follows^40^:

$$AIC = 2n\ln (RSS) + 2param$$

$$RSS = \sum \left( {V_{i}^{real} - \widehat{{V_{i} }}} \right)^{2}$$

where $param$ is the number of parameters in the model. In the full model, it was equal to five (i.e., $\beta_{0}$, $\beta_{1}$, $\beta_{2}$, $\beta_{3}$, $W_{n}$); in the Bayesian learning model, it was equal to four (i.e., $\beta_{0}$, $\beta_{1}$, $\beta_{2}$, $\beta_{3}$). *n* is the number of the candidate reviews (which is equal to 100). A smaller value of the AIC indicates that the distribution generated by the simulation model was closer to the distribution in the real data.

## Supplementary Information

Supplementary Information.

## Data Availability

The raw data analysed in the current study are available at the following URL as an open dataset^24^: https://nijianmo.github.io/amazon/index.html.

## Code availability

The Python code created for the statistical analyses and simulations, as well as the data for making the figures in the current study, are available in a dedicated OSF repository: https://osf.io/8qvuy/?view_only=c2e559ef0d52422f8de2ad364bfd1db2.
