# Supplementary material for: Social influence makes outlier opinions in online reviews offer more helpful information
Source: Sci Rep. 2023 Jun 27;13:9625. doi: 10.1038/s41598-023-35953-4 (PMC10300035; doi:10.1038/s41598-023-35953-4)
Supplement: Supplementary file 1 — Supplementary Information. [file 41598_2023_35953_MOESM1_ESM.pdf]

Supplementary Information

Social influence makes outlier opinions in online reviews offer more helpful information

Kunhao Yang*, Itsuki Fujisaki, Kazuhiro Ueda*

*Kunhao Yang, Kazuhiro Ueda

Email: yangkunhao93@gmail.com, ueda@g.ecc.u-tokyo.ac.jp

This PDF file includes:

Supplementary text

Figures S1 to S14

Tables S1 to S6

Supplementary Information Text

S1. MAD method to identify outlier reviews.

As explained in the manuscript, previous research suggests that z-score can cause bias in detecting outliers when there are extreme values in the dataset (e.g., 1, 1, 2, 6, 1000). Instead, the median absolute deviation is more robust (hereinafter, the *MAD* method). The median absolute deviation is calculated as follows:

$$MAD^{(n)} = b \cdot M_i(|x_i^{(n)} - M_j(x_j^{(n)})|$$

$$OD_i^{(n)} = \frac{|x_i^{(n)} - M_j\left(x_j^{(n)}\right)|}{MAD}$$

where *b* is a constant, the value was set as 1.4626 in this research according to previous research (cited as reference No. 25 in the manuscript); $x_i^{(n)}$ and $x_j^{(n)}$ are the ratings of review *i* and *j* attached to a product *n*. M_i and M_j are calculations to obtain the median rating of product *n*.

To test the robustness of the results reported in the manuscript, we employed the MAD method to measure the outlier degree of reviews. However, the rating can only comprise a narrow range of integer values (one to five on Amazon.com), and the ratings of a certain product often follow a J-shaped distribution. Thus, we found that the MADs of most reviews were zero (i.e., 'Books': 74.21% of the reviews; 'Clothing, Shoes, and Jewellery': 71.36% of the reviews; 'Electronics': 69.87% of the reviews; 'Tools and Home Improvement': 81.80% of the reviews; 'Cell Phones and Accessories': 69.73% of the reviews; 'Automotive': 85.40% of the reviews; 'Sports and Outdoors': 79.56% of the reviews). In the above formula, MAD is used as the denominator to calculate the outlier degree (i.e., $OD_i^{(n)}$). Therefore, the values of zero indicate that the data distribution was

so skewed that MAD method recognised all the values which was not equal to the median as outliers. Thus, MAD method provided a binary rather than a continuous classification of outlier reviews. In this sense, it failed to provide the opportunity for us to analyse the difference in reviews regarding ratings that varied but were not equal to the median rating. However, the values of zero in MADs also implied that it could be too much strict to only focus on the reviews with large (e.g., the top 25%) absolute z-scores. Reviews with a relatively small (e.g., less than one) z-scores could also be considered as outlier reviews by MAD method. Therefore, we examined whether our conclusions in the manuscript were still supported when using the MAD method, which is a more flexible way to define outlier reviews. Reviews whose corresponding median absolute deviations were zeros were divided into either the outlier or the normal groups, referring to whether their ratings were equal to the medians. For reviews whose corresponding median absolute deviations were not zero, we set a threshold as two to divide them into the outlier and normal groups. In other words, when a review's *OD* based on the MAD method was larger than two, it was identified as an outlier review.

We then used the *Mann-Whitney U test* to determine whether the number of votes (i.e., the helpfulness) received by these two groups differed. The results in Fig. S1 show that the reviews in the outlier group received significantly more votes. Additionally, we employed a regression model with the same control variables as the regression model reported in the manuscript. However, we replaced the independent variable (i.e., the outlier degree computed by the z-score method in the original regression model) with a dummy variable indicating whether the review is in the outlier group. The regression results (shown in Tables S4 and S5) also supported our previous results demonstrating

that the reviews in the outlier group received significantly more votes even if controlling for other related variables. Next, we compared the information quality between the outlier and normal groups using the Mann-Whitney U test. As shown in Fig. S2–S4, the outlier group has a higher information quality. Namely, these reviews provided a significantly larger amount of neutral information more concisely.

-----Figure S1 about here-----

-----Figure S2 about here-----

-----Figure S3 about here-----

-----Figure S4 about here-----

S2. Statistical information regarding variables in the negative binomial regression and hierarchical linear regression models.

In the manuscript, negative binomial regression and hierarchical linear regression models were used to examine the relationship between outlier degree and the number of votes. The statistical information regarding the dependent variable, the independent variable, and the control variables in the two regression models is shown in Table S1 and the correlations between these variables are shown in Fig. S5–S11.

Additionally, most reviews with smaller outlier degrees expressed positive opinions, but some reviews with larger outlier degrees expressed negative ones. Therefore, we compared the rating and content positivity distributions between normal and outlier reviews. Both the normal and outlier reviews were identified by the MAD method explained in section ‘S1’. Fig S12 has eight panels displayed in four rows and two columns. For the first seven panels, a bar-plot and a violin-plot were contained in each

panel, which were drawn based on the datasets for the corresponding categories. Each bar-plot and violin-plot shows the distribution of the rating positivity and content positivity of normal and outlier reviews in different categories, respectively. The last panel shows the average number of stars and the average content positivity of reviews with different outlier degrees (i.e., measured based on absolute z-score method in the manuscript) across categories. As shown in Fig. S12, only approximately 1% of the outlier reviews gave five stars (a particularly positive rating). However, this proportion was as high as over 84% in normal reviews. In addition, as the violin plots show, few outlier reviews expressed fully positive opinions (the content positivity is equal to one); by contrast, this proportion is considerable in normal reviews.

-----Table S1 about here-----

-----Figure S5 about here-----

-----Figure S6 about here-----

-----Figure S7 about here-----

-----Figure S8 about here-----

-----Figure S9 about here-----

-----Figure S10 about here-----

-----Figure S11 about here-----

-----Figure S12 about here-----

S3. Regression coefficients of the negative binomial regressions and the hierarchical linear regressions.

As explained in the manuscript, we built negative binomial and hierarchical linear regression models to examine the relationship between outlier degrees and the number of votes. Additionally, as shown in Fig. S5–S11, the correlations between control variables were fairly high. Therefore, we added Lasso regularisation to all our regression models to prevent multicollinearity. With the Lasso regularisation, a regression model attempts to find the smallest model using the fewest variables to fit the dependent variables. Thus, when a variable has collinearity with other variables, the regression coefficients thereof will be set to zero; only variables that independently affect the dependent variable have non-zero regression coefficients. Therefore, the LASSO regularisation effectively prevents multicollinearity among variables. When using LASSO regularization, besides the regression coefficients, *lambda* as the penalty parameter of the LASSO regularisation was also estimated. As lambda increases, the model becomes stricter towards unnecessary variables; that is, more variables will be removed from the model (i.e., the coefficients will be set to zero). The best lambdas reported in the following tables were estimated using the AIC.

The regression coefficients and standard errors of the negative binomial and hierarchical linear regressions using the absolute z-score to measure outlier degrees are shown in Tables S2 and S3, respectively. Meanwhile, the regression coefficients and standard errors of the negative binomial regressions and hierarchical linear regressions using MAD to measure outlier degrees are shown in Tables S4 and S5, respectively.

The coefficients in black are statistically significant, with *p*-values smaller than 0.05, while the coefficients in grey have *p*-values greater than or equal to 0.05.

-----Table S2 about here-----

-----Table S3 about here-----

-----Table S4 about here-----

-----Table S5 about here-----

S4. Cross-correlation coefficients between the distributions of votes obtained from the simulation results and those found in the real data

To demonstrate the robustness of the results shown in Table 2 in the manuscript, we ran additional simulations with different numbers of readers: 5,000, 10,000, or 20,000; Table S6 shows the cross-correlation coefficients, KLDs, and AICs resulting from the different simulation models. The results were consistent with those in Table 2.

-----Table S6 about here-----

S5. High information quality of reviews with low outlier degrees.

In addition to the high information quality of outlier reviews, Fig. 3 in the manuscript also indicates that reviews with small outlier degrees (i.e., around 5 percentile) had higher information quality than reviews with a middle outlier degree (i.e., around 50 percentile). This is especially apparent considering their information entropy and conciseness. To explain this result, we considered that the reviewers' experiences may play an important role.

Fig. S13 (a) shows the relationship between the percentile of outlier degrees of reviews and their author's experiences (i.e., the average number of reviews posted by the corresponding reviewers). We found that the reviewers who posted reviews with outlier degrees around the 5 percentile had the largest average number of reviews across all the categories. This result implied that reviews with small outlier degrees (around 5 percentile) can have a higher information quality than those with middle outlier degrees (around 50 percentile), because more experienced reviewers posted them. However, it was unclear why these reviews generated by experienced reviewers failed to receive more votes (as shown in Fig. 2(b)). We considered that the information quality could also explain this result. When people read reviews, instead of evaluating them by each of three dimensions, they tended to evaluate and vote for the reviews by considering all three dimensions comprehensively (as described in the simulation model). When we observed the three dimensions of information quality separately, both reviews with small and large outlier degrees seemingly provided equally high-quality information. However, when we considered the three dimensions together, we have seen that the information quality of outlier reviews was particularly high. In Fig. S13 (b), we comprehensively measured the information quality by a metric: how many bits (i.e., reflecting sufficiency) of neutral information (i.e., reflecting subjectivity) were provided per word (i.e., reflecting conciseness). The metric was calculated as follows:

$$InQ = \frac{Information\ entropy \cdot (1 - subjectivity)}{length\ of\ the\ review}$$

Using this metric, we found that compared to other reviews, those with large outlier degrees provided significantly high-quality information.

In addition, when considering reviewers' experience, the reviews with the largest

outlier degrees were averagely posted by reviewers with less experience. This result also supported our conclusion regarding the normative social influence mechanism. The outlier reviewers faced a kind of peer pressure which forced them to provide evidence to support their own divergent opinions. Thus, even if they had less experience, they could still provide information with a fairly high quality. Furthermore, this result highlights an interesting issue for future studies to investigate: Why do reviewers with less experience have more chances to post outlier reviews? Previous study (cited as Reference No. 41 in the manuscript) of innovation demonstrated that people with less experience could have more opportunities to achieve higher performances in creative activities. If we consider writing outlier reviews as a kind of creative activity, the above pattern could be more thoroughly explained.

-----Figure S13 about here-----

S6. Month with more outlier reviews.

As mentioned in the "Discussion" section in the manuscript, the mechanism of generating outlier reviews is an interesting avenue for future studies to explore. In this section, we conducted a supplementary analysis based on this suggestion and analysed how many outlier reviews were generated per month in a year. The results are shown in Fig. S14. In this figure, we used the methods based on both the absolute z-score (as explained in the manuscript) and MAD to divide reviews into outlier reviews and normal reviews. When using the absolute z-score, we considered reviews with outlier degrees larger than one as outlier reviews. We then investigated the percentage of outlier reviews across months.

The results based on both two methods were consistent. We found that outlier reviews were more prevalent from June to July and November to December than other periods across all the categories. However, the patterns of change differed slightly between categories.

The above common pattern may be interpreted by the number of reviews posted per month. We found that people posted relatively fewer reviews on Amazon.com in June, July, November, and December. Based on this result, the social influence mechanism can be used to explain the above pattern. Specifically, when outlier reviewers faced relatively few reviews with ratings close to the average, they may feel less peer pressure to post an outlier review. Consequently, there were more outlier reviews from June to July and November to December.

However, understanding the above common pattern requires more analysis; further, it could be caused by random fluctuations. The grey areas (95% CIs) in Fig. S14 indicate that the change in likelihood of outlier reviews among months was statistically significant. However, these changes were negligible (approximately 0.5%). As people posted relatively fewer reviews in June, July, November, and December, random fluctuations could have a larger impact on the likelihood of outlier reviews in these months than in other months. Therefore, our present results cannot support a solid conclusion about the mechanism of the above pattern, and future research is encouraged to further explore this issue.

-----Figure S14 about here-----

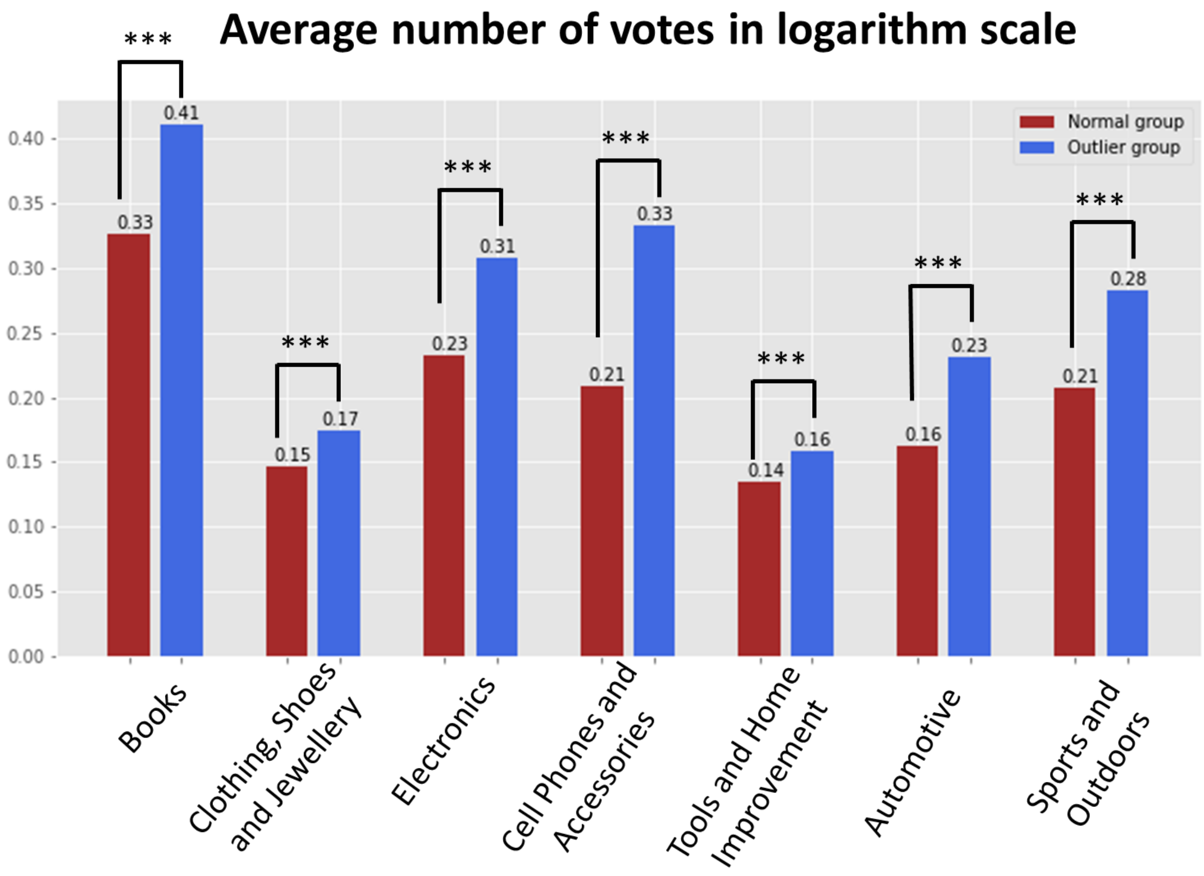

Fig. S1. Average number of votes in logarithm scale between the outlier group and the normal group across all categories. The outlier group and the normal group were identified according to MAD method. The asterisk indicates the statistical significance of difference between the two groups through Mann-Whitney U test. Three asterisks indicate that the *p-value* is less than 0.001. Note that the error bars reflecting standard errors are too narrow to be seen. Therefore, they are omitted in the figure.

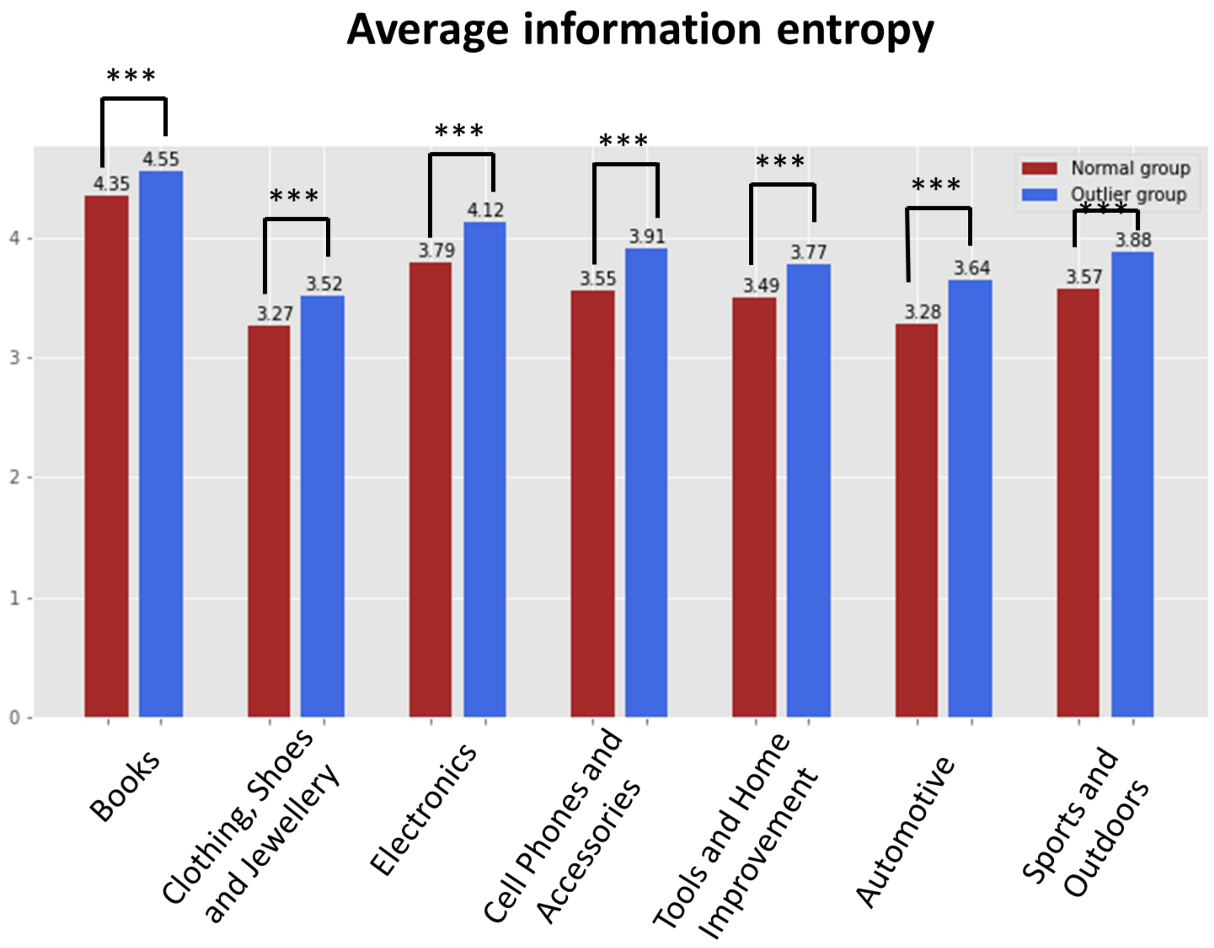

Fig. S2. Average information entropy between the outlier group and the normal group across all categories. The outlier group and the normal group were identified according to MAD method. The asterisk indicates the statistical significance of difference between the two groups through Mann-Whitney U test. Three asterisks indicate that the *p-value* is less than 0.001. Note that the error bars reflecting standard errors are too narrow to be seen. Therefore, they are omitted in the figure.

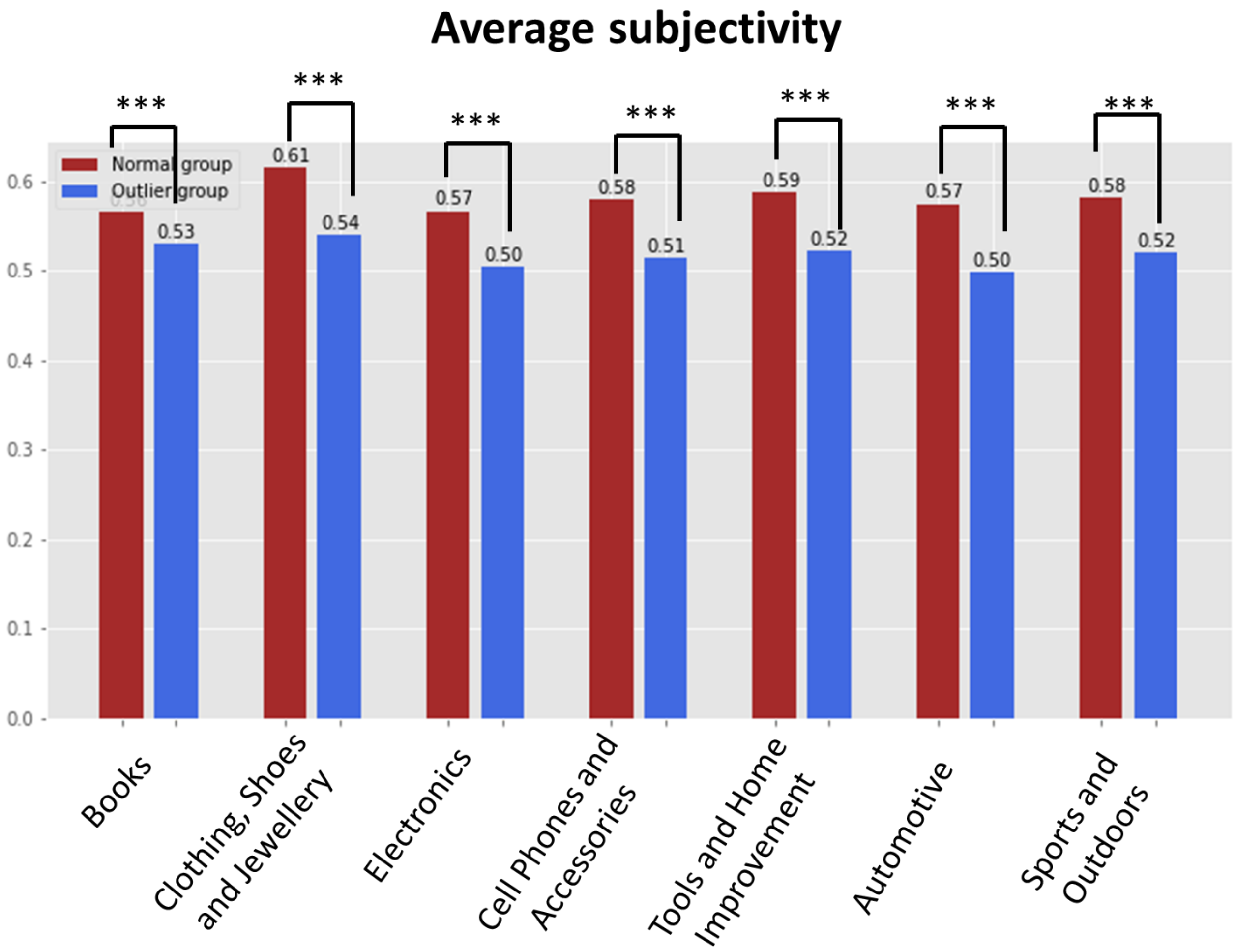

Fig. S3. Average subjectivity between the outlier group and the normal group across all categories. The outlier group and the normal group were identified according to MAD method. The asterisk indicates the statistical significance of difference between the two groups through Mann-Whitney U test. Three asterisks indicate that the *p-value* is less than 0.001. Note that the error bars reflecting standard errors are too narrow to be seen. Therefore, they are omitted in the figure.

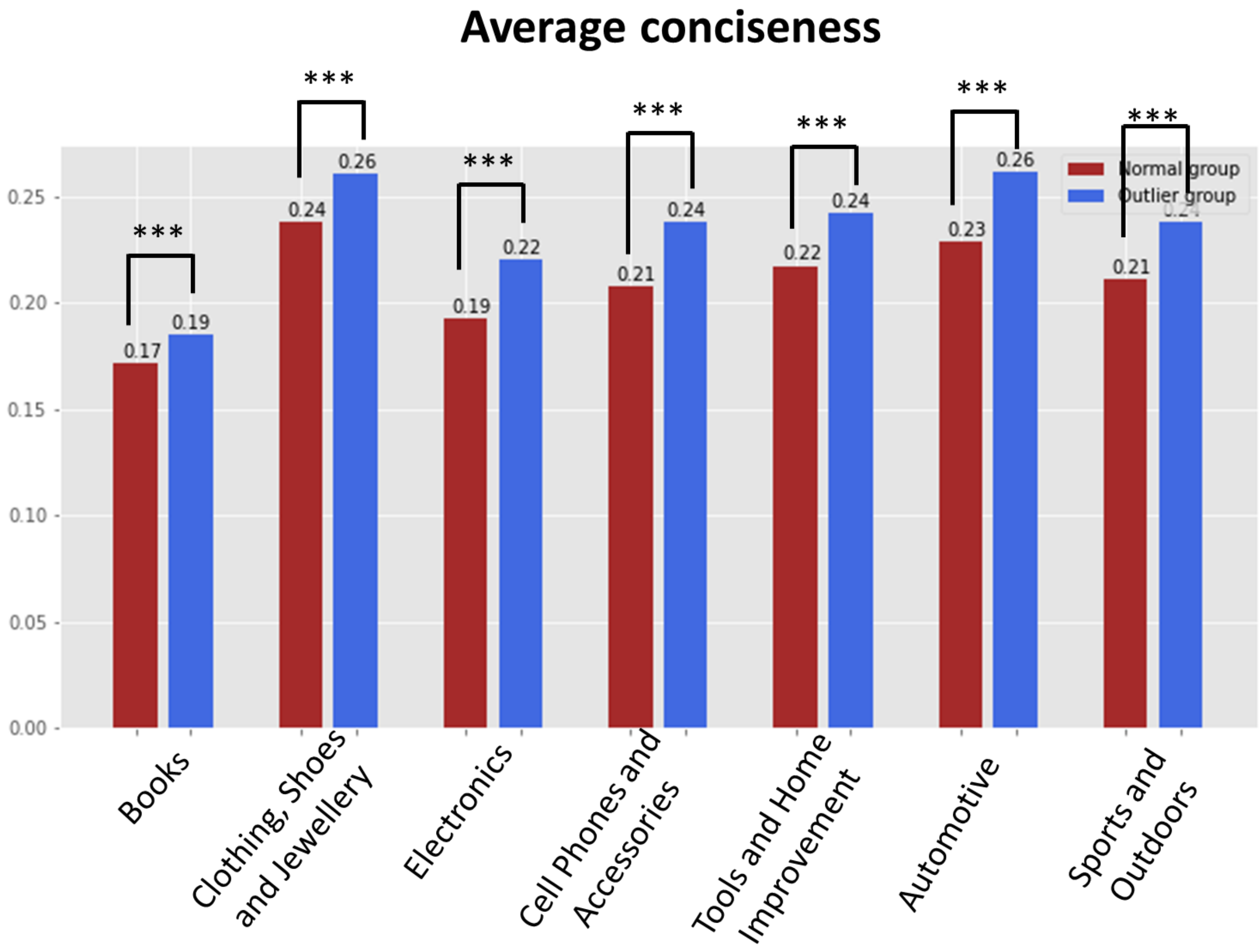

Fig. S4. Average conciseness between the outlier group and the normal group across all categories. The outlier group and the normal group were identified according to MAD method. The asterisk indicates the statistical significance of difference between the two groups through Mann-Whitney U test. Three asterisks indicate that the *p-value* is less than 0.001. Note that the error bars reflecting standard errors are too narrow to be seen. Therefore, they are omitted in the figure.

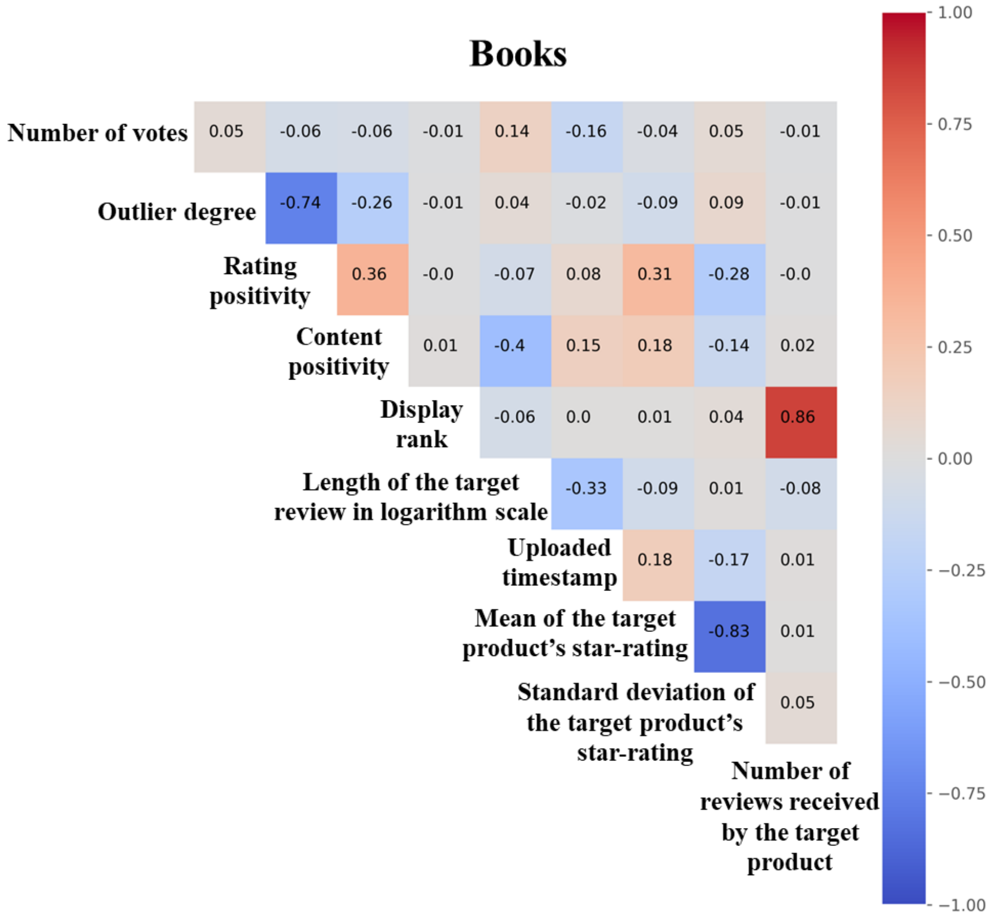

Fig. S5. The correlations among the variables in the regression model reported in the manuscript. The correlation coefficients are shown in a matrix with the names of the variables in the diagonal. The colour and number of every element in the matrix shows the values of the correlation coefficients. All coefficients were statistically significant with *p*-values ≤ 0.01. The product category of the data is shown above the matrix.

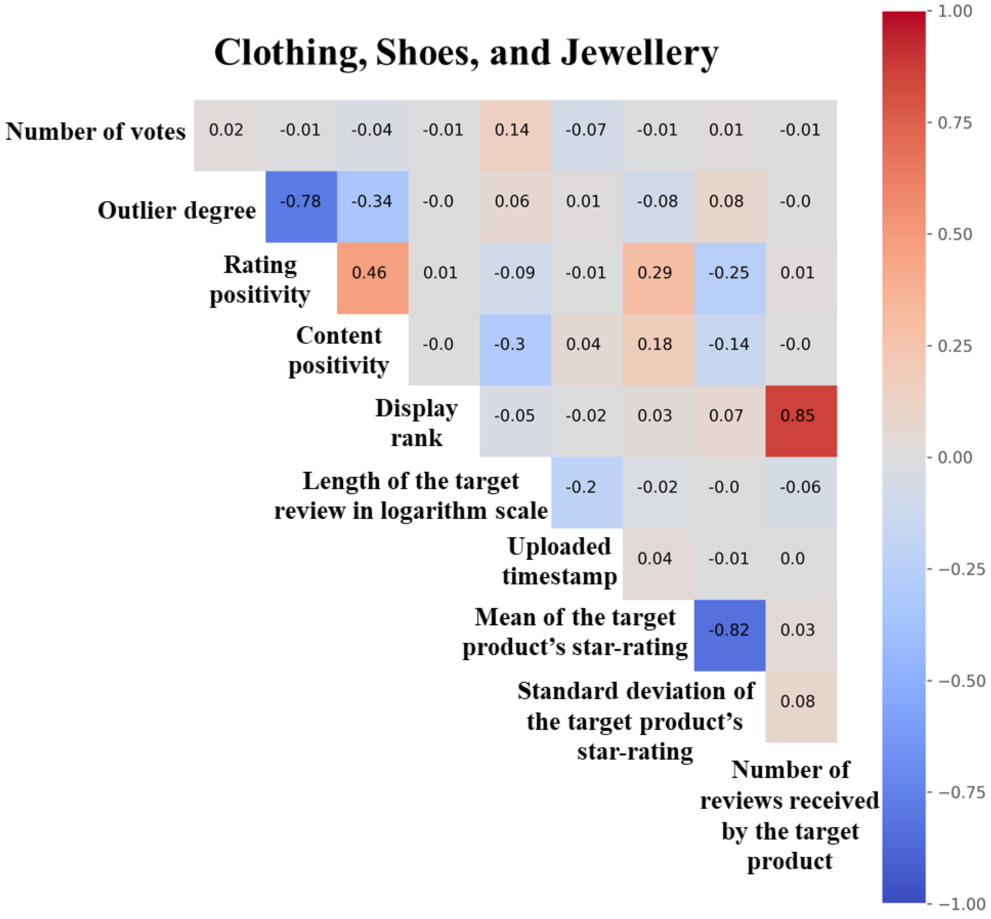

Fig. S6. The correlations among the variables in the regression model reported in the manuscript. The correlation coefficients are shown in a matrix with the names of the variables in the diagonal. The colour and number of every element in the matrix shows the values of the correlation coefficients. All coefficients were statistically significant with *p*-values ≤ 0.01. The product category of the data is shown above the matrix.

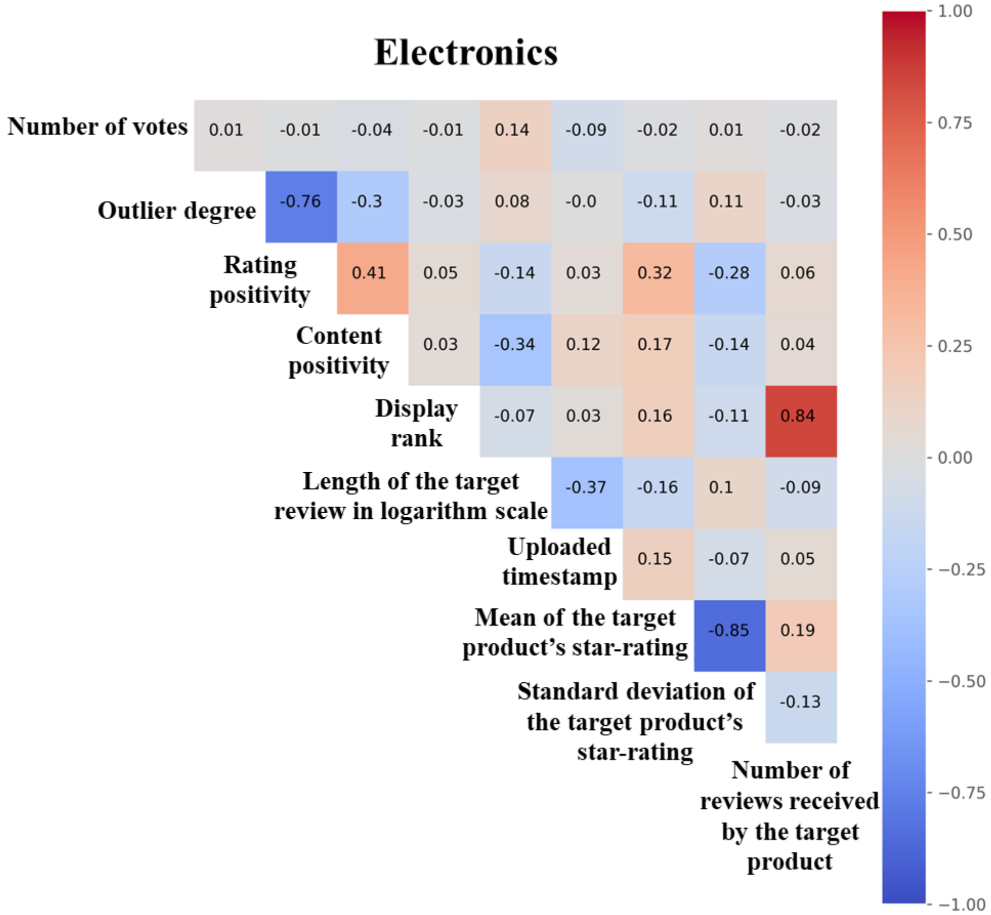

Fig. S7. The correlations among the variables in the regression model reported in the manuscript. The correlation coefficients are shown in a matrix with the names of the variables in the diagonal. The colour and number of every element in the matrix shows the values of the correlation coefficients. All coefficients were statistically significant with *p*-values ≤ 0.01. The product category of the data is shown above the matrix.

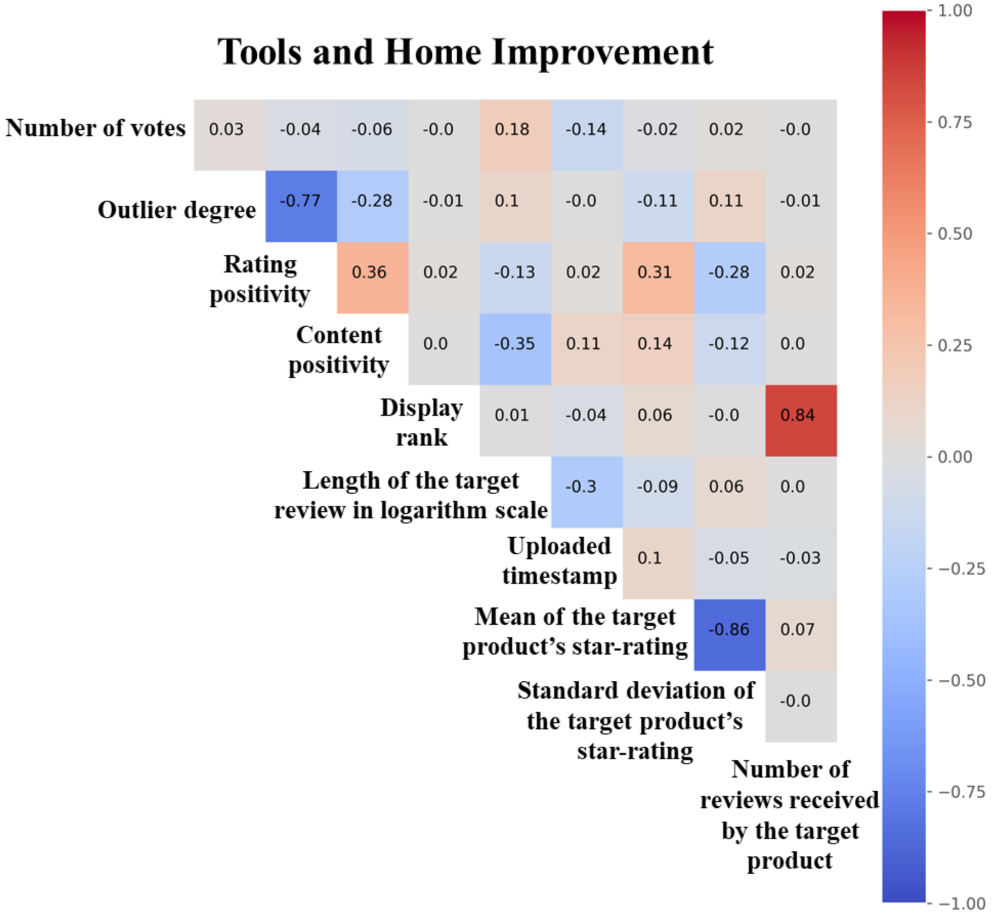

Fig. S8. The correlations among the variables in the regression model reported in the manuscript. The correlation coefficients are shown in a matrix with the names of the variables in the diagonal. The colour and number of every element in the matrix shows the values of the correlation coefficients. All coefficients were statistically significant with p-values ≤ 0.01. The product category of the data is shown above the matrix.

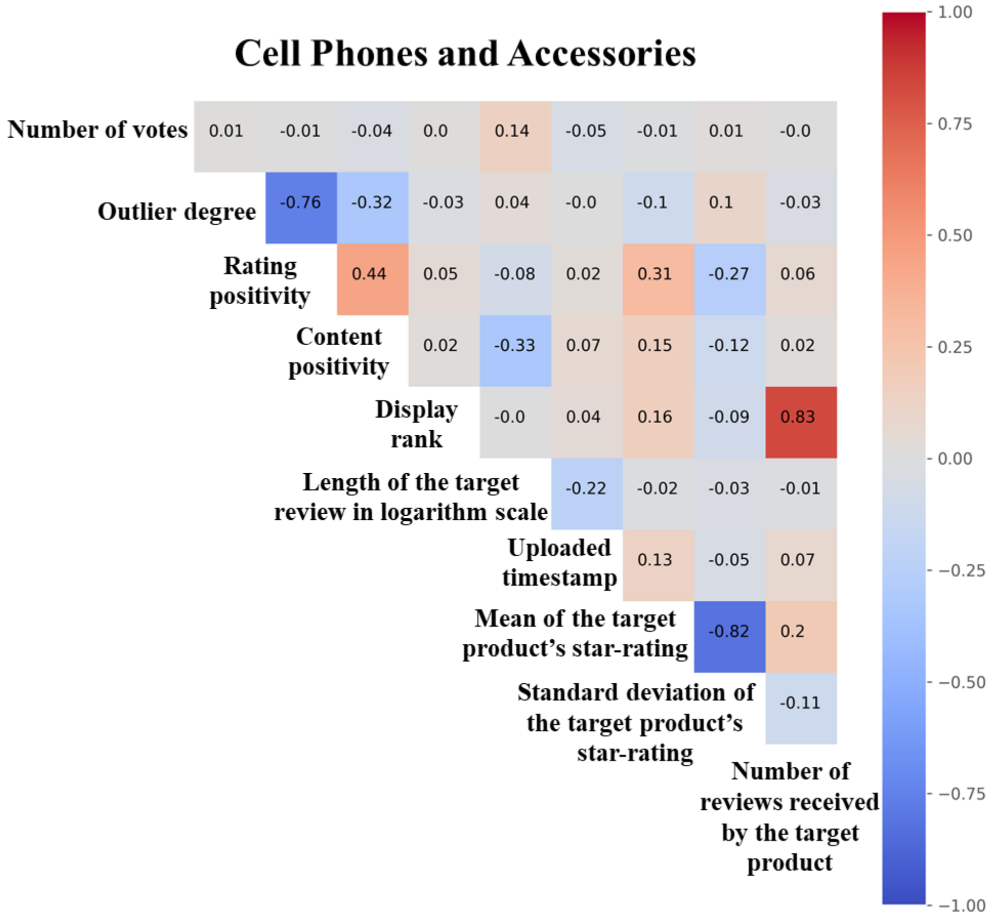

Fig. S9. The correlations among the variables in the regression model reported in the manuscript. The correlation coefficients are shown in a matrix with the names of the variables in the diagonal. The colour and number of every element in the matrix shows the values of the correlation coefficients. All coefficients were statistically significant with *p*-values ≤ 0.01. The product category of the data is shown above the matrix.

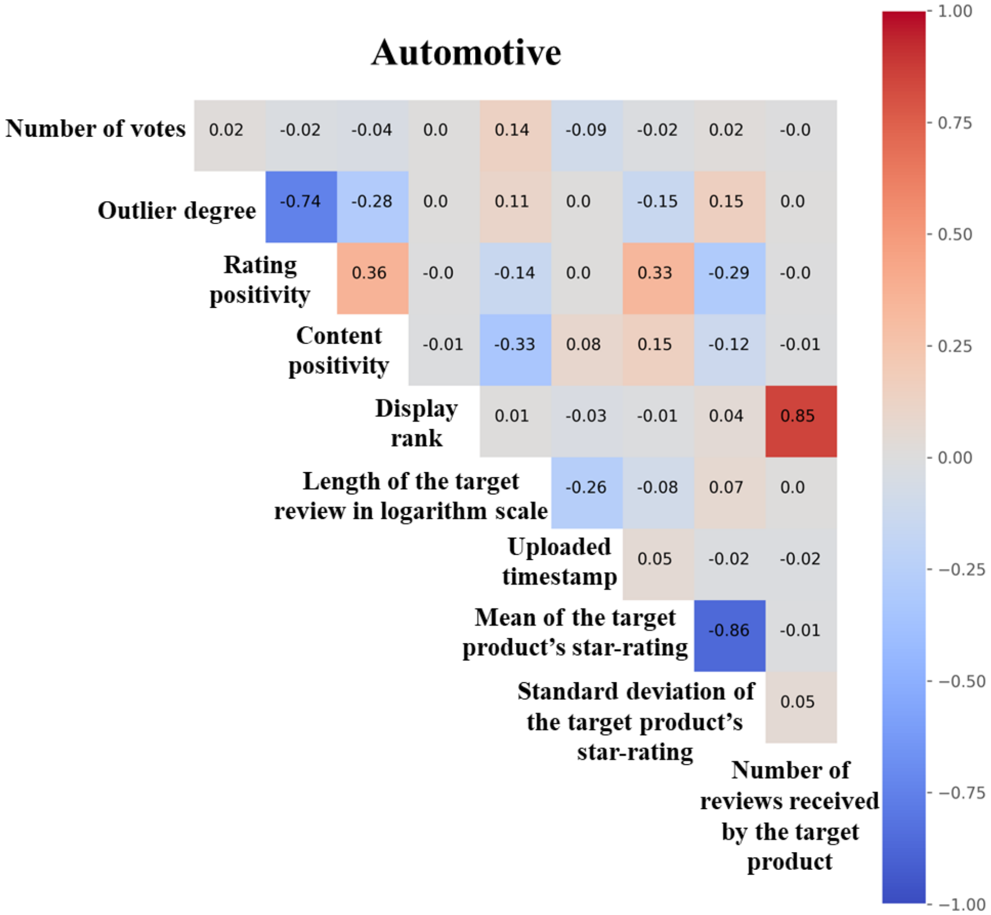

Fig. S10. The correlations among the variables in the regression model reported in the manuscript. The correlation coefficients are shown in a matrix with the names of the variables in the diagonal. The colour and number of every element in the matrix shows the values of the correlation coefficients. All coefficients were statistically significant with p-values ≤ 0.01. The product category of the data is shown above the matrix.

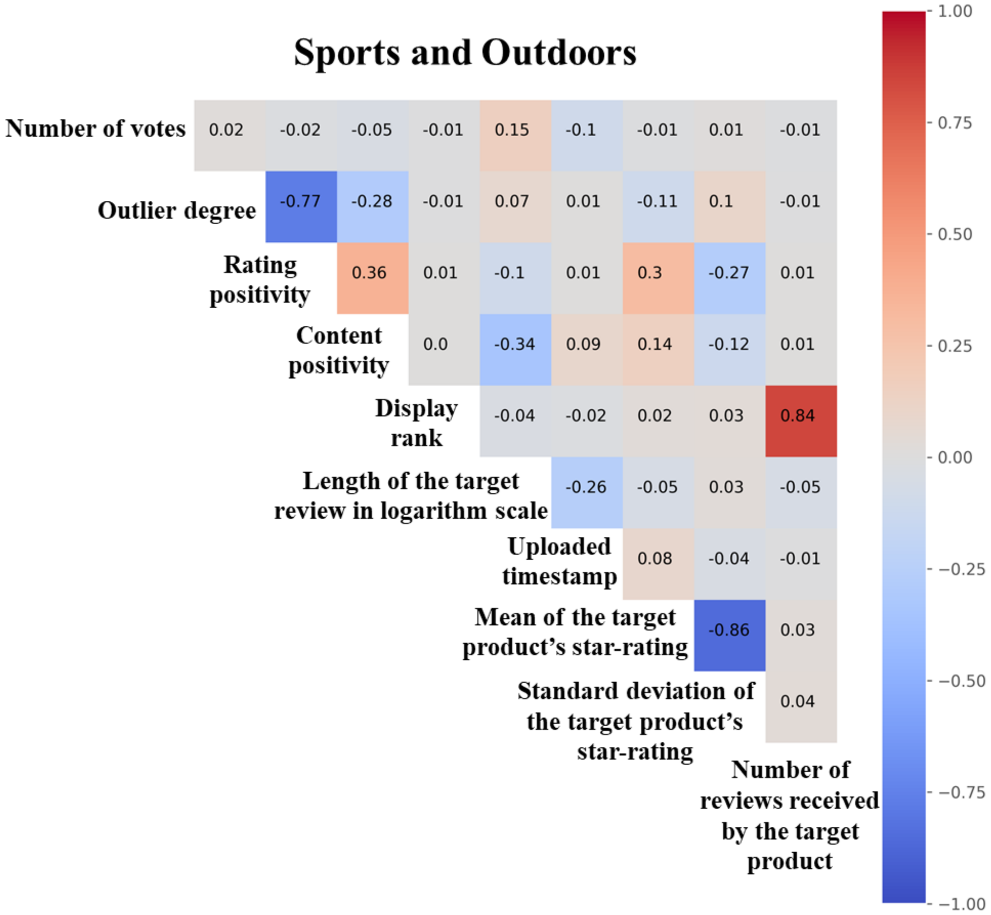

Fig. S11. The correlations among the variables in the regression model reported in the manuscript. The correlation coefficients are shown in a matrix with the names of the variables in the diagonal. The colour and number of every element in the matrix shows the values of the correlation coefficients. All coefficients were statistically significant with *p*-values ≤ 0.01. The product category of the data is shown above the matrix.

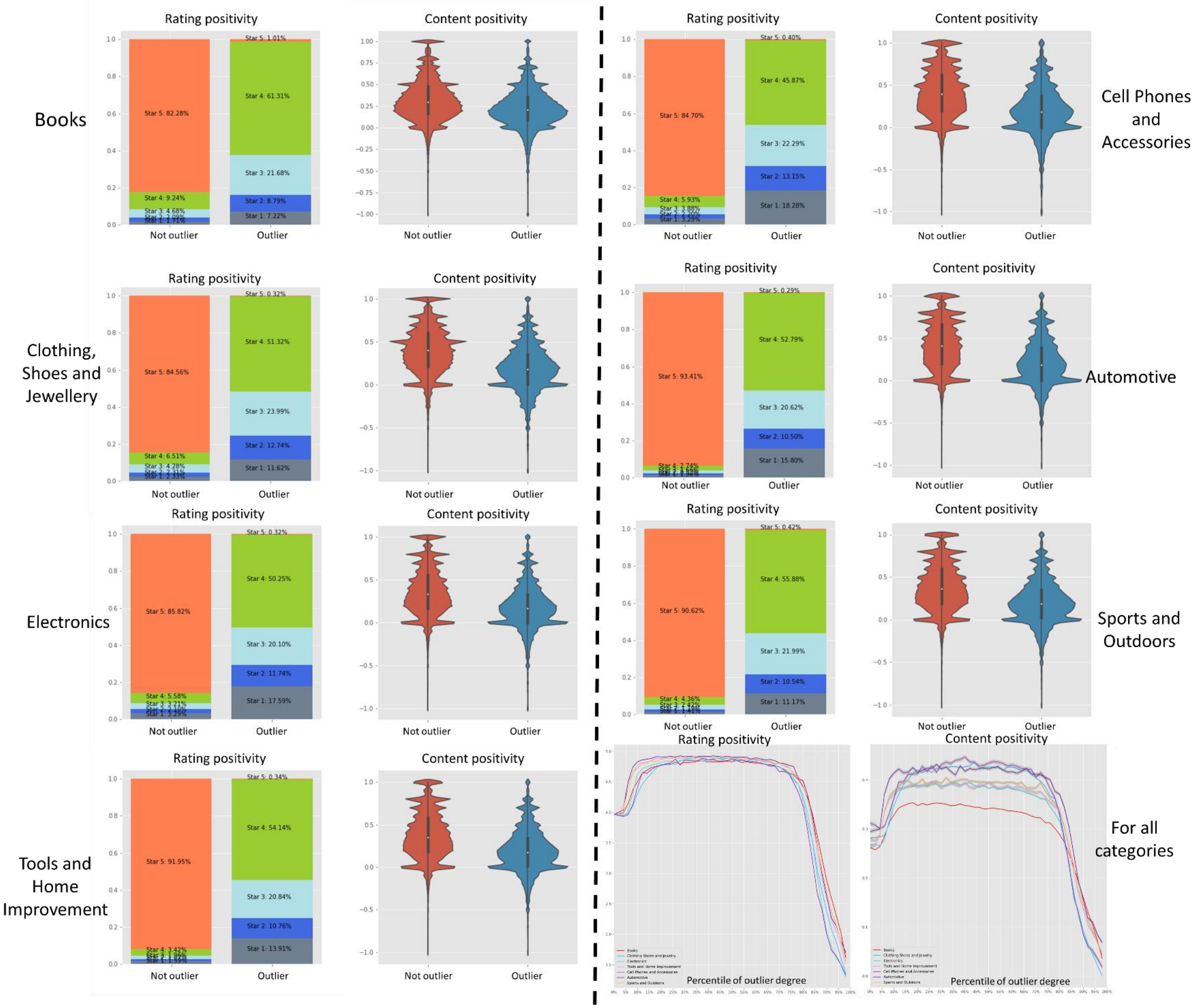

Fig. S12. Positivity distributions between outlier reviews and normal reviews. Eight panels were displayed in four rows and two columns. In the first seven panels, a bar-plot and a violin-plot were contained in each panel, which were drawn based on the datasets of various categories. Each bar-plot shows the distribution of the rating positivity of normal reviews and outlier reviews in each of the different categories. Each violin-plot shows the distribution of the content positivity of normal reviews and outlier review in each of the different categories. The last panel shows the average number of stars and the

average content positivity of reviews with different outlier degrees based on the absolute z-score across different categories.

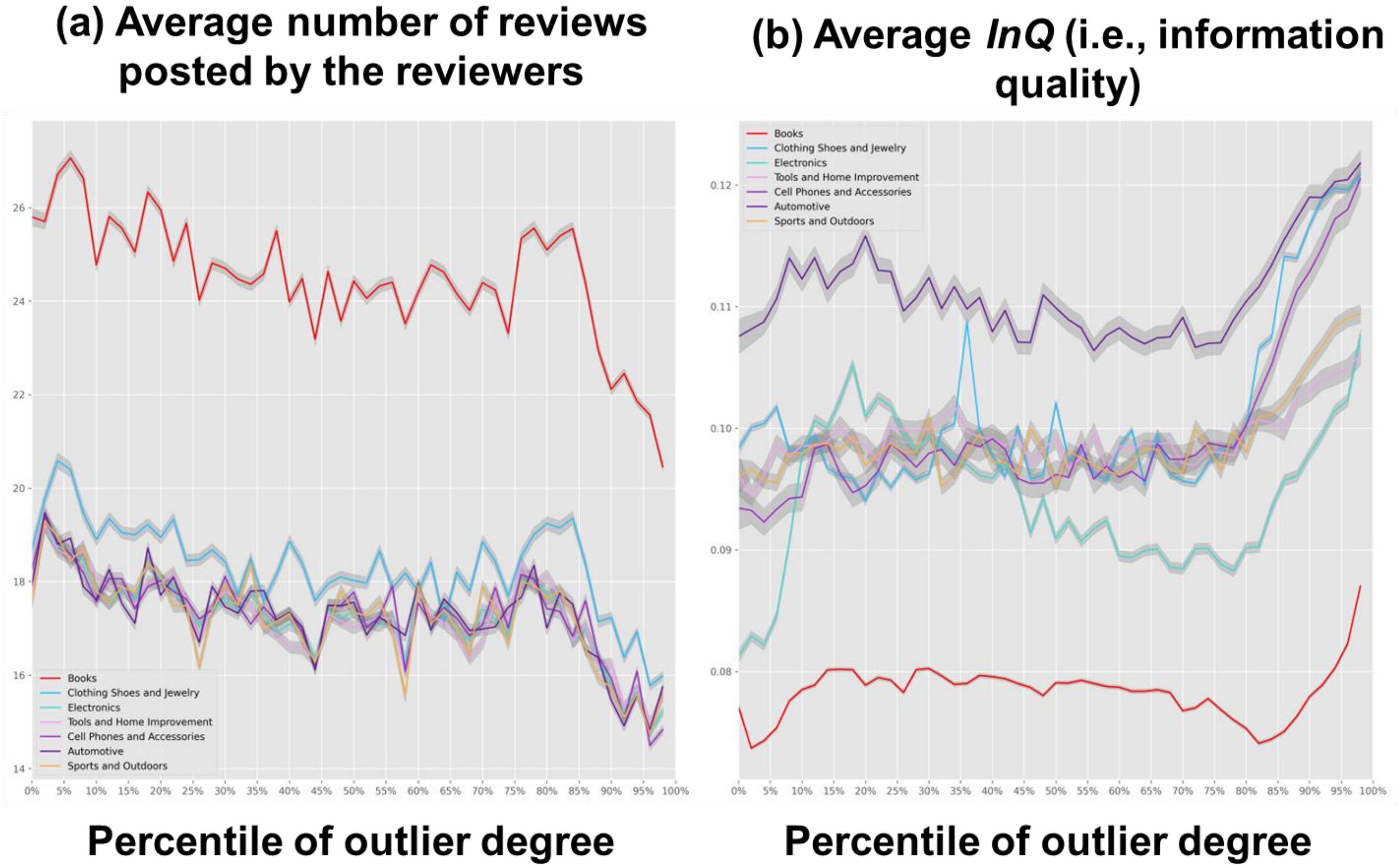

Fig. S13. Fig. S13 (a) shows the average number of reviews posted by the corresponding reviewers of the reviews with different outlier degrees. The x-axis represents the percentile of the outlier degree for a review. The y-axis represents the average experience of reviewers. The colours of lines reflect the results for reviews in different product categories. Fig. S13 (b) shows the average information quality of reviews with different outlier degrees. The x-axis represents the percentile of the outlier degree for a review. The y-axis represents how many bits of neutral information was provided per word in each review. As in Fig. S13 (a), the different colours of the lines reflect the results based on the reviews in different product categories.

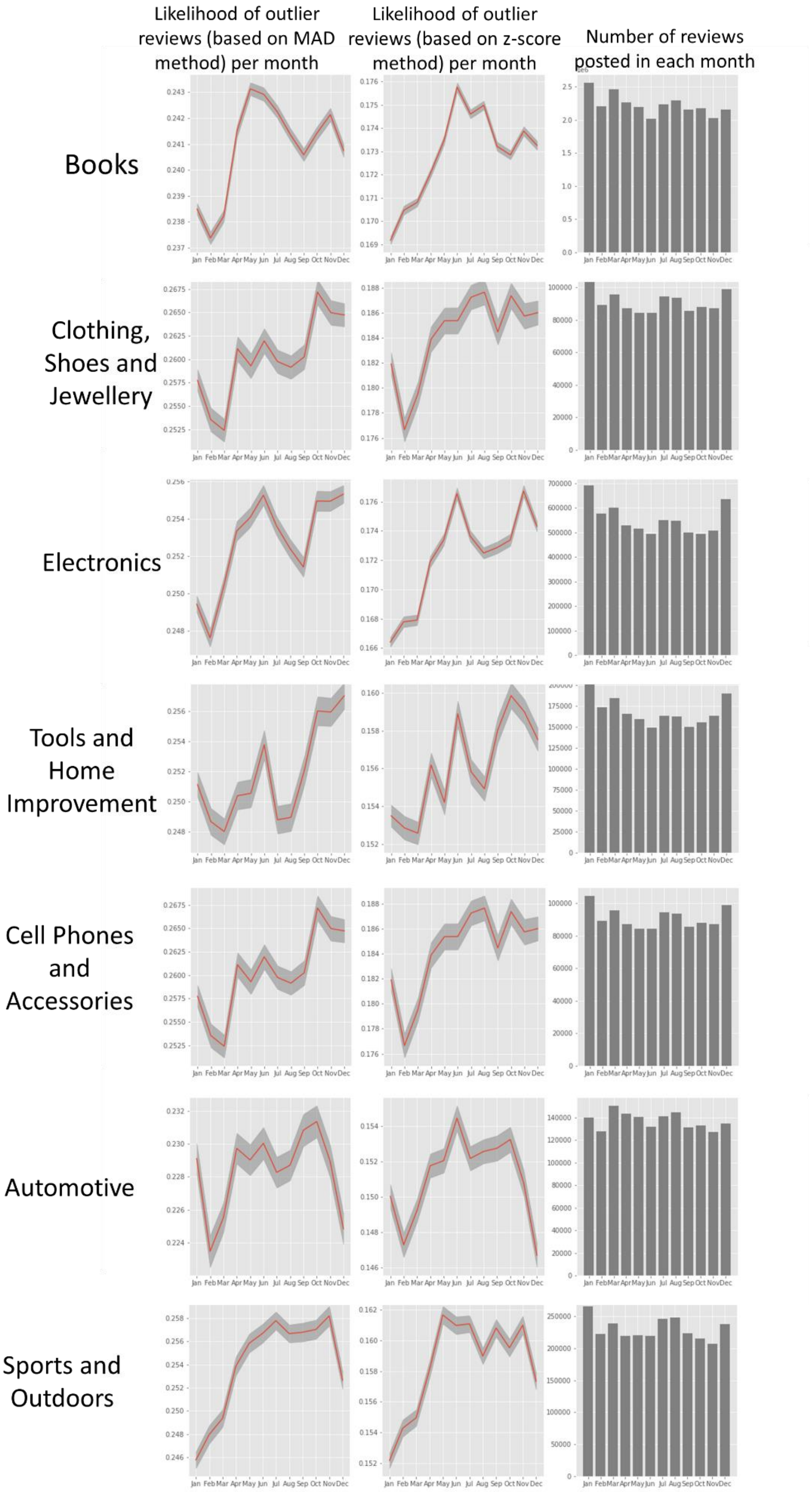
Likelihood of outlier reviews (based on MAD method) per month
Likelihood of outlier reviews (based on z-score method) per month
Number of reviews posted in each month
Books
Clothing, Shoes and Jewellery
Electronics
Tools and Home Improvement
Cell Phones and Accessories
Automotive
Sports and Outdoors

Fig. S14. Seven panels reflecting the results based on different categories were included. Each panel includes three plots. The plot at left shows the likelihood of outlier degrees per month based on MAD method; the one at middle shows the likelihood of outlier degrees per month based on absolute z-score method. The plot at right shows the number of reviews being posted per month.

Table S1. Statistical information regarding the variables in the hierarchical linear regression model found in the manuscript.

Name	Count	Mean	Std. Dev.	Min	Max
Category: Books					
**Name**	**Count**	**Mean**	**Std. Dev.**	**Min**	**Max**
Dependent Variable					
Number of votes	27,164,983	1.76	15.19	0	19,391
Independent Variable					
Outlier degree	27,164,983	0.77	0.64	0.00	13.71
Control Variables					
Rating positivity	27,164,983	0.78	0.55	-1	1
Content positivity	27,164,983	0.30	0. 25	-1	1
Display rank	27,164,983	379.65	1385.49	0	28,974
Length of the target review in logarithm scale	27,164,983	3.34	1.07	0.00	8.14
Uploaded timestamp	27,164,983	20141676.97	31826.90	19960520.00	20181002.00
Mean of the target product's rating	27,164,983	4.37	0.36	1	5
Standard deviation of the target product's rating	27,164,983	0.90	0.28	0.00	2.19
Number of reviews received by the target product	27,164,983	760.29	2369.43	6	28,975
Information Quality					
Information entropy	27,164,983	4.40	1.47	0.00	11.48
Subjectivity	27,164,983	0.56	0.17	0.00	0.47
Conciseness	25,044,376	0.29	0.10	0.11	1.47
Category: Clothing, Shoes, and Jewellery					
**Name**	**Count**	**Mean**	**Std. Dev.**	**Min**	**Max**
Dependent Variable					
Number of votes	11,285,464	0.64	6.23	0	2,330
Independent Variable					
Outlier degree	11,285,464	0.78	0.62	0	8.83
Control Variables					
Rating positivity	11,285,464	0.71	0.64	-1	1
Content positivity	11,285,464	0.35	0.29	-1	1
Display rank	11,285,464	380.16	1239.93	0	19,692
Length of the target review in logarithm scale	11,285,464	2.6	0.81	0	7.95
Uploaded timestamp	11,285,464	20156790	14638.11	20030323	20181005

Mean of the target product's rating	11,285,464	4.28	0.36	1	5
Standard deviation of the target product's rating	11,285,464	1.05	0.28	0	2.19
Number of reviews received by the target product	11,285,464	761.33	2113.61	6	19,693
Information Quality					
Information entropy	11,285,464	3.33	1.16	0	8.61
Subjectivity	11,285,464	0.59	0.22	0	1
Conciseness	9,102,113	0.37	0.09	0.15	1.42
Category: Electronics					
**Name**	**Count**	**Mean**	**Std. Dev.**	**Min**	**Max**
Dependent Variable					
Number of votes	6,739,590	1.58	20.12	0	9,096
Independent Variable					
Outlier degree	6,739,590	0.76	0.65	0	11.58
Control Variables					
Rating positivity	6,739,590	0.7	0.66	-1	1
Content positivity	6,739,590	0.32	0.29	-1	1
Display rank	6,739,590	279.41	640.51	0	8,616
Length of the target review in logarithm scale	6,739,590	3	1.04	0	8.11
Uploaded timestamp	6,739,590	20147186	23237.88	19990613	20181004
Mean of the target product's Rating	6,739,590	4.27	0.41	1	5
Standard deviation of the target product's rating	6,739,590	1.1	0.31	0	2.19
Number of reviews received by the target product	6,739,590	559.82	1073.51	6	8,617
Information Quality					
Information entropy	6,739,590	3.88	1.43	0	10.6
Subjectivity	6,739,590	0.55	0.22	0	1
Conciseness	5,112,499	0.35	0.10	0.15	1.42
Category: Tools and Home Improvement					
**Name**	**Count**	**Mean**	**Std. Dev.**	**Min**	**Max**
Dependent Variable					
Number of votes	2,070,831	1.17	10.65	0	2,427
Independent Variable					

Outlier degree	2,070,831	0.74	0.66	0	9.33
Control Variables					
Rating positivity	2,070,831	0.77	0.58	-1	1
Content positivity	2,070,831	0.33	0.29	-1	1
Display rank	2,070,831	106.67	255.87	0	3,870
Length of the target review in logarithm scale	2,070,831	2.83	0.96	0	8.04
Uploaded timestamp	2,070,831	20154483	18395.24	19991108	20181004
Mean of the target product's rating	2,070,831	4.41	0.36	1	5
Standard deviation of the target product's rating	2,070,831	0.97	0.33	0	2.19
Number of reviews received by the target product	2,070,831	214.35	430.03	6	3,871
Information Quality					
Information entropy	2,070,831	3.64	1.34	0	8.98
Subjectivity	2,070,831	0.56	0.22	0	1
Conciseness	1,804,707	0.36	0.10	0.15	1.29
Category: Cell Phones and Accessories					
**Name**	**Count**	**Mean**	**Std. Dev.**	**Min**	**Max**
Dependent Variable					
Number of votes	1,128,437	0.78	10.22	0	2,038
Independent Variable					
Outlier degree	1,128,437	0.78	0.62	0	10.82
Control Variables					
Rating positivity	1,128,437	0.67	0.68	-1	1
Content positivity	1,128,437	0.35	0.31	-1	1
Display rank	1,128,437	70.58	144.78	0	1,683
Length of the target review in logarithm scale	1,128,437	2.79	1	0	8.05
Uploaded timestamp	1,128,437	20152237	14686.4	20021023	20181002
Mean of the target product's rating	1,128,437	4.22	0.4	1	5
Standard deviation of the target product's rating	1,128,437	1.14	0.32	0	2.19
Number of reviews received by the target product	1,128,437	142.16	240.47	6	1,684
Information Quality					

Information entropy	1,128,437	3.57	1.38	0	9.52
Subjectivity	1,128,437	0.57	0.23	0	1
Conciseness	977,310	0.35	0.10	0.14	1.38
Category: Automotive					
**Name**	**Count**	**Mean**	**Std. Dev.**	**Min**	**Max**
Dependent Variable					
Number of votes	1,711,519	0.67	7.86	0	5,853
Independent Variable					
Outlier degree	1,711,519	0.71	0.68	0	9.88
Control Variables					
Rating positivity	1,711,519	0.79	0.57	-1	1
Content positivity	1,711,519	0.37	0.31	-1	1
Display rank	1,711,519	81.04	233.97	0	5,060
Length of the target review in logarithm scale	1,711,519	2.62	0.87	0	7.48
Uploaded timestamp	1,711,519	20156573	15859.9	20000914	20181003
Mean of the target product's rating	1,711,519	4.46	0.38	1	5
Standard deviation of the target product's rating	1,711,519	0.93	0.39	0	2.19
Number of reviews received by the target product	1,711,519	163.07	396.96	6	5,061
Information Quality					
Information entropy	1,711,519	3.36	1.24	0	8.57
Subjectivity	1,711,519	0.56	0.24	0	1
Conciseness	1,569,821	0.39	0.10	0.17	1.32
Category: Sports and Outdoors					
**Name**	**Count**	**Mean**	**Std. Dev.**	**Min**	**Max**
Dependent Variable					
Number of votes	2,839,940	1.09	11.28	0	4,862
Independent Variable					
Outlier degree	2,839,940	0.75	0.65	0	11.31
Control Variables					
Rating positivity	2,839,940	0.78	0.57	-1	1
Content positivity	2,839,940	0.34	0.29	-1	1
Display rank	2,839,940	109.55	263.12	0	4,162

Length of the target review in logarithm scale	2,839,940	2.82	0.93	0	8.05
Uploaded timestamp	2,839,940	20154253	16581.72	20000915	20181003
Mean of the target product's rating	2,839,940	4.42	0.34	1	5
Standard deviation of the target product's rating	2,839,940	0.94	0.32	0	2.19
Number of reviews received by the target product	2,839,940	220.1	442.26	6	4,163
Information Quality					
Information entropy	2,839,940	3.65	1.3	0	9.16
Subjectivity	2,839,940	0.57	0.22	0	1
Conciseness	2,010,940	0.37	0.10	0.15	1.45

Table S2. The regression coefficients of the negative binomial model found in the manuscript.

**Category:**	**Books**	
	**Coefficient**	**Standard Error**
	**Independent Variable**	
Outlier degree	0.0508^{***}	0.0008
	**Control Variables**	
Rating positivity	-0.3263^{***}	0.0009
Content positivity	-0.1065^{***}	0.0002
Display rank	0.0159^{***}	0.0013
Length of the target review in logarithm scale	1.0873^{***}	0.0007
Uploaded timestamp	-0.5664^{***}	0.0006
Mean of the target product's rating	0.2639^{***}	0.0008
Standard deviation of the target product's rating	0.3792^{***}	0.0008
Number of reviews received by the target product	-0.2227^{***}	0.0013
Intercept	-0.6237^{***}	0.0006
Alpha	7.2819^{***}	0.0045
Lambda	0.0014	/
**Count of data points: 27,164,983**	**Pseudo R-squared: 0.092**	**AIC: 52754321.8023**

**Category:**	**Clothing, Shoes, and Jewellery**	
	**Coefficient**	**Standard Error**
	**Independent Variable**	
Outlier degree	0.2386^{***}	0.0023
	**Control Variables**	
Rating positivity	0.1405^{***}	0.0026
Content positivity	-0.0199^{***}	0.0018

Display rank	0.0087**	0.0030
Length of the target review in logarithm scale	1.1853***	0.0016
Uploaded timestamp	-0.3298***	0.0015
Mean of the target product's rating	-0.0437***	0.0026
Standard deviation of the target product's rating	0.1365***	0.0025
Number of reviews received by the target product	-0.2178***	0.0030
Intercept	-1.3857***	0.0015
alpha	17.4696***	0.0231
lambda	0.0013	/
**Count of data points: 11,285,464**	**Pseudo R-squared: 0.061**	**AIC: 11917506.8151**
**Category:**	**Electronics**	
	**Coefficient**	**Standard Error**
**Independent Variable**		
Outlier degree	0.2532***	0.0023
**Control Variables**		
Rating positivity	0.0111***	0.0025
Content positivity	-0.0694***	0.0020
Display rank	0.0378***	0.0032
Length of the target review in logarithm scale	1.4840***	0.0018
Uploaded timestamp	-0.3947***	0.0014
Mean of the target product's rating	0.0516***	0.0027
Standard deviation of the target product's rating	0.1031***	0.0026

Number of reviews received by the target product	-0.3934***	0.0033
Intercept	-1.1620***	0.0006
alpha	10.2174***	0.0144
lambda	0.0011	/
**Count of data points: 6,739,590**	**Pseudo R-squared: 0.102**	**AIC: 10066053.4067**
**Category:**	**Tools and Home Improvement**	
	**Coefficient**	**Standard Error**
	**Independent Variable**	
Outlier degree	0.1702***	0.0040
	**Control Variables**	
Rating positivity	-0.1082***	0.0044
Content positivity	-0.0632***	0.0034
Display rank	-0.0116***	0.0051
Length of the target review in logarithm scale	1.4436***	0.0032
Uploaded timestamp	-0.4925***	0.0026
Mean of the target product's rating	0.0690***	0.0050
Standard deviation of the target product's rating	0.0624***	0.0049
Number of reviews received by the target product	-0.2472***	0.0051
Intercept	-1.3350***	0.0029
alpha	8.7418***	0.0231
lambda	0.0015	/
**Count of data points: 2,070,831**	**Pseudo R-squared: 0.108**	**AIC: 2939829.0051**
**Category:**	**Cell Phones and Accessories**	
	**Coefficient**	**Standard Error**

**Independent Variable**		
Outlier degree	0.2997***	0.0078
**Control Variables**		
Rating positivity	0.1016***	0.0088
Content positivity	-0.0284***	0.0066
Display rank	0.0236**	0.0095
Length of the target review in logarithm scale	1.4417***	0.0056
Uploaded timestamp	-0.2005***	0.0051
Mean of the target product's rating	0.0918***	0.0086
Standard deviation of the target product's rating	0.1768***	0.0084
Number of reviews received by the target product	-0.2872***	0.0096
Intercept	-1.6161***	0.0053
alpha	20.2548***	0.0909
lambda	0.0025	
**Count of data points: 1,128,437**	**Pseudo R-squared: 0.077**	**AIC: 1060857.5633**

**Category:**	**Automotive**	
	**Coefficient**	**Standard Error**
**Independent Variable**		
Outlier degree	0.1017***	0.0046
**Control Variables**		
Rating positivity	-0.0864***	0.0050
Content positivity	-0.0405***	0.0040
Display rank	0.0336***	0.0075
Length of the target review in logarithm scale	1.3636***	0.0037
Uploaded timestamp	-0.4484***	0.0031

Mean of the target product's rating	0.0164^{***}	0.0062
Standard deviation of the target product's rating	0.0837^{***}	0.0062
Number of reviews received by the target product	-0.2944^{***}	0.0078
Intercept	-1.7314^{***}	0.0035
alpha	9.4633^{***}	0.0322
lambda	0.001	/
**Count of data points: 1,711,519**	**Pseudo R-squared: 0.107**	**AIC: 1913981.5432**

**Category:**	**Sports and Outdoors**	
	**Coefficient**	**Standard Error**
**Independent Variable**		
Outlier degree	0.1907^{***}	0.0036
**Control Variables**		
Rating positivity	-0.0065	0.0040
Content positivity	-0.0438^{***}	0.0030
Display rank	0.0166^{***}	0.0048
Length of the target review in logarithm scale	1.3802^{***}	0.0027
Uploaded timestamp	-0.4309^{***}	0.0023
Mean of the target product's rating	0.0746^{***}	0.0044
Standard deviation of the target product's rating	0.1348^{***}	0.004
Number of reviews received by the target product	-0.2669^{***}	0.0049
Intercept	-1.2526^{***}	0.0025
alpha	10.1935^{***}	0.0234
lambda	0.0002	/
**Count of data points: 2,839,940**	**Pseudo R-squared: 0.094**	**AIC: 3911730.2268**

Note: All variables were standardized (i.e., $x_{new}^{i} = \frac{x^{i} - mean(x^{j})}{std.dev(x^{j})}$); the significance levels of the coefficients were indicated by asterisks; * $p < 0.1$, ** $p < 0.05$, *** $p < 0.01$.

Table S3. The regression coefficients of the hierarchical linear regressions found in the manuscript.

**Category:**	**Books**	
	**Coefficient**	**Standard Error**
	**Independent Variable**	
Outlier degree	0.0307***	0.0002
	**Control Variables**	
Rating positivity	0.1050***	0.0002
Content positivity	0.0128***	0.0001
Display rank	-0.0053***	0.0002
Length of the target review in logarithm scale	0.2178***	0.0001
Mean of the target product's rating	0.1099***	0.0002
Standard deviation of the target product's rating	0.1244***	0.0002
Number of reviews received by the target product	-0.0418***	0.0002
Lambda	0.0014	/
**Count of data points: 27,164,983**	**Pseudo R-squared: 0.296**	**AIC: 53804676.3920**
**Category:**	**Clothing, Shoes, and Jewellery**	
	**Coefficient**	**Standard Error**
	**Independent Variable**	
Outlier degree	0.0282***	0.0002
	**Control Variables**	
Rating positivity	0.0089***	0.0003
Content positivity	0.0052***	0.0002
Display rank	-0.0021***	0.0003

Length of the target review in logarithm scale	0.1623***	0.0002
Mean of the target product's rating	-0.0007**	0.0003
Standard deviation of the target product's rating	0.0127***	0.0003
Number of reviews received by the target product	-0.0213***	0.0003
lambda	0.0013	/
**Count of data points: 11,285,464**	**Pseudo R-squared: 0.115**	**AIC: 15647508.1953**
**Category:**	**Electronics**	
	**Coefficient**	**Standard Error**
	**Independent Variable**	
Outlier degree	0.0434***	0.0004
	**Control Variables**	
Rating positivity	-0.0061***	0.0004
Content positivity	0.0153***	0.0003
Display rank	-0.0056***	0.0004
Length of the target review in logarithm scale	0.2610***	0.0003
Mean of the target product's rating	0.0097***	0.0005
Standard deviation of the target product's rating	0.0057***	0.0005
Number of reviews received by the target product	-0.0391***	0.0004
lambda	0.0011	/
**Count of data points: 6,739,590**	**Pseudo R-squared:** 0.218	**AIC:** 12519599.0347
**Category:**	**Tools and Home Improvement**	

	Coefficient	Standard Error
**Independent Variable**		
Outlier degree	0.0357***	0.0007
**Control Variables**		
Rating positivity	-0.0272***	0.0007
Content positivity	0.0074***	0.0005
Display rank	-0.0059***	0.0008
Length of the target review in logarithm scale	0.2408***	0.0005
Mean of the target product's rating	0.0172***	0.0008
Standard deviation of the target product's rating	0.0088***	0.0008
Number of reviews received by the target product	-0.0433***	0.0008
lambda	0.0015	/
**Count of data points: 2,070,831**	**Pseudo R-squared:** 0.228	**AIC:** 3544576.7186
**Category:**	**Cell Phones and Accessories**	
	**Coefficient**	**Standard Error**
**Independent Variable**		
Outlier degree	0.0320***	0.0007
**Control Variables**		
Rating positivity	0.0136***	0.0008
Content positivity	0.0090***	0.0006
Display rank	0.0003	0.0009
Length of the target review in logarithm scale	0.1793***	0.0005
Mean of the target product's rating	0.0088***	0.0009

Standard deviation of the target product's rating	0.0248***	0.0008
Number of reviews received by the target product	-0.0214***	0.0009
lambda	0.0025	
**Count of data points: 1,128,437**	**Pseudo R-squared: 0.123**	**AIC: 1566500.5164**
**Category:**	**Automotive**	
	**Coefficient**	**Standard Error**
**Independent Variable**		
Outlier degree	0.0128***	0.0006
**Control Variables**		
Rating positivity	-0.0102***	0.0006
Content positivity	0.0013***	0.0004
Display rank	-0.0035***	0.0007
Length of the target review in logarithm scale	0.1997***	0.0004
Mean of the target product's rating	0.0050***	0.0008
Standard deviation of the target product's rating	0.0071***	0.0008
Number of reviews received by the target product	-0.0227***	0.0007
lambda	0.001	/
**Count of data points: 1,711,519**	**Pseudo R-squared: 0.190**	**AIC: 2324119.7783**
**Category:**	**Sports and Outdoors**	
	**Coefficient**	**Standard Error**
**Independent Variable**		
Outlier degree	0.0342***	0.0006

Control Variables		
Rating positivity	-0.0053***	0.0006
Content positivity	0.0058***	0.0004
Display rank	-0.0039***	0.0006
Length of the target review in logarithm scale	0.2365***	0.0004
Mean of the target product's rating	0.0200***	0.0007
Standard deviation of the target product's rating	0.0220***	0.0007
Number of reviews received by the target product	-0.0327***	0.0006
lambda	0.0002	/
**Count of data points: 2,839,940**	**Pseudo R-squared: 0.190**	**AIC: 4803190.7602**

Note: All variables were standardized (i.e., $x_{new}^{i} = \frac{x^{i} - mean(x^{j})}{std.dev(x^{j})}$); the significance levels of the coefficients were indicated by asterisks; * p < 0.1, ** p < 0.05, *** p < 0.01.

Table S4. The regression coefficients of the negative binomial model using a dummy variable that reflect whether a review is identified to be an outlier review by MAD method as the independent variable.

**Category:**	**Books**	
	**Coefficient**	**Standard Error**
**Independent Variable**		
Dummy: if is an outlier review	0.3275***	0.0016
**Control Variables**		
Rating positivity	-0.4254***	0.0007
Content positivity	-0.1166***	0.0008
Display rank	0.0143***	0.0013
Length of the target review in logarithm scale	1.0900***	0.0007
Uploaded timestamp	-0.5667***	0.0006
Mean of the target product's rating	0.3116***	0.0008
Standard deviation of the target product's rating	0.4006***	0.0008
Number of reviews received by the target product	-0.2222***	0.0013
Intercept	-0.5515***	0.0007
Alpha	7.2492***	0.0044
Lambda	0.0013	/
**Count of data points: 27,164,983**	**Pseudo R-squared: 0.090**	**AIC: 52717116.1456**
**Category:**	**Clothing, Shoes, and Jewellery**	
	**Coefficient**	**Standard Error**
**Independent Variable**		
Dummy: if is an outlier review	0.2168***	0.0040
**Control Variables**		

Rating positivity	-0.1121***	0.0018
Content positivity	-0.0318***	0.0019
Display rank	0.0078**	0.0030
Length of the target review in logarithm scale	1.1842***	0.0016
Uploaded timestamp	-0.3282***	0.0015
Mean of the target product's rating	0.0352***	0.0026
Standard deviation of the target product's rating	0.1656***	0.0026
Number of reviews received by the target product	-0.2100***	0.0030
Intercept	-1.3184***	0.0018
alpha	17.5777***	0.0233
lambda	0.0013	/
**Count of data points: 11,285,464**	**Pseudo R-squared: 0.061**	**AIC: 11925546.5986**
**Category:**	**Electronics**	
	**Coefficient**	**Standard Error**
	**Independent Variable**	
Dummy: if is an outlier review	0.1343***	0.0041
	**Control Variables**	
Rating positivity	-0.2403***	0.0018
Content positivity	-0.0750***	0.0020
Display rank	0.0368***	0.0032
Length of the target review in logarithm scale	1.4803***	0.0018
Uploaded timestamp	-0.3957***	0.0014
Mean of the target product's rating	0.1312***	0.0027

Standard deviation of the target product's rating	0.1314***	0.0027
Number of reviews received by the target product	-0.3831***	0.0033
Intercept	-1.1134***	0.0019
alpha	10.3077***	0.0145
lambda	0.0011	/
**Count of data points: 6,739,590**	**Pseudo R-squared: 0.101**	**AIC: 10077802.9568**
**Category:**	**Tools and Home Improvement**	
	**Coefficient**	**Standard Error**
	**Independent Variable**	
Dummy: if is an outlier review	0.0850***	0.0070
	**Control Variables**	
Rating positivity	-0.2777***	0.0031
Content positivity	-0.0715***	0.0035
Display rank	-0.0112***	0.0051
Length of the target review in logarithm scale	1.4405***	0.0032
Uploaded timestamp	-0.4926***	0.0026
Mean of the target product's rating	0.1203***	0.0051
Standard deviation of the target product's rating	0.0785***	0.0050
Number of reviews received by the target product	-0.2428***	0.0050
Intercept	-1.3081***	0.0034
alpha	8.7794***	0.0232
lambda	0.0015	/

Count of data points: 2,070,831	Pseudo R-squared: 0.108	AIC: 2941551.6797
**Category:**	**Cell Phones and Accessories**	
	**Coefficient**	**Standard Error**
	**Independent Variable**	
Dummy: if is an outlier review	0.3734***	0.0143
	**Control Variables**	
Rating positivity	-0.2507***	0.0065
Content positivity	-0.0421***	0.0066
Display rank	0.0252***	0.0095
Length of the target review in logarithm scale	1.4356***	0.0056
Uploaded timestamp	-0.2053***	0.0051
Mean of the target product's rating	0.2190***	0.0088
Standard deviation of the target product's rating	0.2266***	0.0085
Number of reviews received by the target product	-0.2698***	0.0096
Intercept	-1.5053***	0.0064
alpha	20.4255***	0.0915
lambda	0.0025	

Count of data points: 1,128,437	Pseudo R-squared: 0.077	AIC: 1061802.7159
**Category:**	**Automotive**	
	**Coefficient**	**Standard Error**
	**Independent Variable**	
Dummy: if is an outlier review	0.2005***	0.0089
	**Control Variables**	
Rating positivity	-0.2140***	0.0037

Content positivity	-0.0512***	0.0040
Display rank	0.0386***	0.0074
Length of the target review in logarithm scale	1.3649***	0.0037
Uploaded timestamp	-0.4489***	0.0031
Mean of the target product's rating	0.0674***	0.0062
Standard deviation of the target product's rating	0.1140***	0.0063
Number of reviews received by the target product	-0.2898***	0.0078
Intercept	-1.6855***	0.0040
alpha	9.4590***	0.0322
lambda	0.001	/
**Count of data points: 1,711,519**	**Pseudo R-squared: 0.107**	**AIC:** 1913975.7778
**Category:**	**Sports and Outdoors**	
	**Coefficient**	**Standard Error**
	**Independent Variable**	
Dummy: if is an outlier review	0.1517***	0.0063
	**Control Variables**	
Rating positivity	-0.2079***	0.0027
Content positivity	-0.0558***	0.0030
Display rank	0.0172***	0.0048
Length of the target review in logarithm scale	1.3779***	0.0027
Uploaded timestamp	-0.4310***	0.0023
Mean of the target product's rating	0.1300***	0.0045
Standard deviation of the target product's rating	0.1527***	0.0045

Number of reviews received by the target product	-0.2602^{***}	0.0049
Intercept	-1.2078^{***}	0.0030
alpha	10.2387^{***}	0.0235
lambda	0.0002	/
**Count of data points: 2,839,940**	**Pseudo R-squared: 0.093**	**AIC: 3914048.3198**

Note: All variables were standardized (i.e., $x_{new}^{i} = \frac{x^{i} - mean(x^{j})}{std.dev(x^{j})}$); the significance levels of the coefficients were indicated by asterisks; * $p < 0.1$, ** $p < 0.05$, *** $p < 0.01$.

Table S5. The regression coefficients of the hierarchical linear regressions using a dummy variable that reflect whether a review is identified to be an outlier review by MAD method as the independent variable.

**Category:**	**Books**	
	**Coefficient**	**Standard Error**
	**Independent Variable**	
Dummy: if is an outlier review	0.0708***	0.0003
	**Control Variables**	
Rating positivity	-0.1398***	0.0002
Content positivity	0.0099***	0.0002
Display rank	-0.0053***	0.0002
Length of the target review in logarithm scale	0.2188***	0.0001
Mean of the target product's rating	0.1261***	0.0002
Standard deviation of the target product's rating	0.1304***	0.0002
Number of reviews received by the target product	-0.0423***	0.0002
Lambda	0.0014	/
**Count of data points: 27,164,983**	**Pseudo R-squared: 0.297**	**AIC: 53782145.3909**
**Category:**	**Clothing, Shoes, and Jewellery**	
	**Coefficient**	**Standard Error**
	**Independent Variable**	
Dummy: if is an outlier review	0.0225***	0.0004
	**Control Variables**	
Rating positivity	-0.0185***	0.0002
Content positivity	0.0041***	0.0002
Display rank	-0.0021***	0.0003

Length of the target review in logarithm scale	0.1624***	0.0002
Mean of the target product's rating	0.0080**	0.0003
Standard deviation of the target product's rating	0.0151***	0.0003
Number of reviews received by the target product	-0.0211***	0.0003
lambda	0.0013	/
**Count of data points: 11,285,464**	**Pseudo R-squared: 0.114**	**AIC: 15657657.6410**
**Category:**	**Electronics**	
	**Coefficient**	**Standard Error**
	**Independent Variable**	
Dummy: if is an outlier review	0.0209***	0.0006
	**Control Variables**	
Rating positivity	-0.0453***	0.0003
Content positivity	0.0142***	0.0003
Display rank	-0.0057***	0.0004
Length of the target review in logarithm scale	0.2610***	0.0003
Mean of the target product's rating	0.0212***	0.0005
Standard deviation of the target product's rating	0.0091***	0.0005
Number of reviews received by the target product	-0.0397***	0.0004
lambda	0.0011	/
**Count of data points: 6,739,590**	**Pseudo R-squared:** 0.216	**AIC:** 12531392.8560

Category:	Tools and Home Improvement	
	Coefficient	Standard Error
	**Independent Variable**	
Dummy: if is an outlier review	0.0041^{***}	0.0011
	**Control Variables**	
Rating positivity	-0.0570^{***}	0.0005
Content positivity	0.0067^{***}	0.0005
Display rank	-0.0059^{***}	0.0008
Length of the target review in logarithm scale	0.2406^{***}	0.0005
Mean of the target product's rating	0.0236^{***}	0.0008
Standard deviation of the target product's rating	0.0100^{***}	0.0008
Number of reviews received by the target product	-0.0434^{***}	0.0008
lambda	0.0015	/
**Count of data points: 2,070,831**	**Pseudo R-squared: 0.227**	**AIC: 3547486.3884**

Category:	Cell Phones and Accessories	
	Coefficient	Standard Error
	**Independent Variable**	
Dummy: if is an outlier review	0.0308^{***}	0.0013
	**Control Variables**	
Rating positivity	-0.0187^{***}	0.0006
Content positivity	0.0077^{***}	0.0006
Display rank	0.0003	0.0009
Length of the target review in logarithm scale	0.1790^{***}	0.0005

Mean of the target product's rating	0.0196***	0.0009
Standard deviation of the target product's rating	0.0281***	0.0008
Number of reviews received by the target product	-0.0221***	0.0009
lambda	0.0025	
**Count of data points: 1,128,437**	**Pseudo R-squared:** 0.122	**AIC:** 1567772.2034
**Category:**	**Automotive**	
	**Coefficient**	**Standard Error**
	**Independent Variable**	
Dummy: if is an outlier review	0.0244***	0.0011
	**Control Variables**	
Rating positivity	-0.0251***	0.0005
Content positivity	-0.0001	0.0004
Display rank	-0.0035***	0.0007
Length of the target review in logarithm scale	0.2000***	0.0004
Mean of the target product's rating	0.0104***	0.0008
Standard deviation of the target product's rating	0.0104***	0.0008
Number of reviews received by the target product	-0.0224***	0.0007
lambda	0.001	/
**Count of data points: 1,711,519**	**Pseudo R-squared: 0.190**	**AIC: 2324094.2938**
**Category:**	**Sports and Outdoors**	
	**Coefficient**	**Standard Error**

**Independent Variable**		
Dummy: if is an outlier review	0.0188***	0.0009
**Control Variables**		
Rating positivity	-0.0366***	0.0004
Content positivity	0.0043***	0.0004
Display rank	-0.0039***	0.0006
Length of the target review in logarithm scale	0.2367***	0.0004
Mean of the target product's rating	0.0275***	0.0007
Standard deviation of the target product's rating	0.0238***	0.0007
Number of reviews received by the target product	-0.0326***	0.0006
lambda	0.0002	/
**Count of data points: 2,839,940**	**Pseudo R-squared: 0.189**	**AIC: 4806499.9161**

Note: All variables were standardized (i.e., $x_{new}^{i} = \frac{x^{i} - mean(x^{j})}{std.dev(x^{j})}$); the significance levels of the coefficients were indicated by asterisks; * $p < 0.1$, ** $p < 0.05$, *** $p < 0.01$.

Table S6. The cross-correlation coefficients, KLDs, and AICs resulting from the different simulation models and different numbers of readers.

Product category	Full model				Bayesian learning model		
	Number of Readers	Cross-correlation coefficient	KLD	AIC	Cross-correlation coefficient	KLD	AIC
Books	5,000	0.86***	0.12	1,228.98	-0.16	0.49	1,244.80
	10,000	0.83***	0.11	1,165.58	-0.16	0.54	1,287.52
	20,000	0.80***	0.13	1,271.96	-0.16	0.58	1,327.48
Clothing, Shoes, and Jewellery	5,000	0.68***	0.17	1,103.99	0.06	0.38	1,232.82
	10,000	0.59***	0.18	1,166.02	0.03	0.42	1,275.43
	20,000	0.59***	0.18	1,166.02	0.00	0.46	1,315.84
Electronics	5,000	0.15*	0.13	851.32	-0.33**	0.49	1,252.51
	10,000	0.14*	0.11	809.42	-0.32**	0.55	1,298.01
	20,000	0.14*	0.11	809.42	-0.36**	0.58	1,331.52
Tools and Home Improvement	5,000	0.45***	0.16	970.08	-0.07	0.44	1,236.33
	10,000	0.34***	0.22	1,055.68	-0.07	0.47	1,273.06
	20,000	0.24***	0.27	1,126.09	-0.10	0.52	1,319.95
Cell Phones and Accessories	5,000	0.28**	0.18	962.41	-0.22**	0.45	1,248.43
	10,000	0.10*	0.31	1,141.10	-0.21**	0.49	1,286.68
	20,000	0.10*	0.31	1,141.10	-0.23**	0.53	1,321.58
Automotive	5,000	0.64***	0.18	1,089.83	0.06	0.38	1,227.89
	10,000	0.50***	0.23	1,213.46	0.09	0.42	1,269.52
	20,000	0.56***	0.20	1,134.73	0.06	0.45	1,307.24
Sports and Outdoors	5,000	0.63***	0.17	1,036.94	0.03	0.40	1,229.57
	10,000	0.51***	0.22	1,111.59	0.03	0.44	1,276.65
	20,000	0.51***	0.22	1,111.59	-0.01	0.47	1,309.27

Note. * $p < 0.1$, ** $p < 0.05$, *** $p < 0.01$.
